# *In vitro* characterization of nonribosomal peptide synthetase-dependent *O*-(2-hydrazineylideneacetyl)serine synthesis indicates a stepwise oxidation strategy to generate the α-diazo ester moiety of azaserine†

**DOI:** 10.1039/d3sc01906c

**Published:** 2023-07-03

**Authors:** Yusuke Shikai, Seiji Kawai, Yohei Katsuyama, Yasuo Ohnishi

**Affiliations:** a Department of Biotechnology, Graduate School of Agricultural and Life Sciences, The University of Tokyo 1-1-1 Yayoi, Bunkyo-ku Tokyo 113-8657 Japan; b Collaborative Research Institute for Innovative Microbiology, The University of Tokyo Bunkyo-ku Tokyo 113-8657 Japan aykatsuhko@g.ecc.u-tokyo.ac.jp

## Abstract

Azaserine, a natural product containing a diazo group, exhibits anticancer activity. In this study, we investigated the biosynthetic pathway to azaserine. The putative azaserine biosynthetic gene (*azs*) cluster, which contains 21 genes, including those responsible for hydrazinoacetic acid (HAA) synthesis, was discovered using bioinformatics analysis of the *Streptomyces fragilis* genome. Azaserine was produced by the heterologous expression of the *azs* cluster in *Streptomyces albus*. *In vitro* enzyme assays using recombinant Azs proteins revealed the azaserine biosynthetic pathway as follows. AzsSPTF and carrier protein (CP) AzsQ are used to synthesize the 2-hydrazineylideneacetyl (HDA) moiety attached to AzsQ from HAA. AzsD transfers the HDA moiety to the C-terminal CP domain of AzsN. The heterocyclization (Cy) domain of the nonribosomal peptide synthetase AzsO synthesizes *O*-(2-hydrazineylideneacetyl)serine (HDA-Ser) attached to its CP domain from l-serine and HDA moiety-attached AzsN. The thioesterase AzsB hydrolyzes it to yield HDA-Ser, which appears to be converted to azaserine by oxidation. Bioinformatics analysis of the Cy domain of AzsO showed that it has a conserved DxxxxD motif; however, two conserved amino acid residues (Thr and Asp) important for heterocyclization are substituted for Asn. Site-directed mutagenesis of two Asp residues in the DxxxxD motif (D193 and D198) and two substituted Asn residues (N414 and N447) indicated that these four residues are important for ester bond synthesis. These results showed that the diazo ester of azasrine is synthesized by the stepwise oxidation of the HAA moiety and provided another strategy to biosynthesize the diazo group.

## Abstract 2

Identification of azaserine biosynthetic gene cluster and *in vitro* reconstitution of *O*-(2-hydrazineylideneacetyl)serine synthesis showed that the diazo group of azaserine is synthesized by the stepwise oxidation of a hydrazinoacetic acid moiety.

## Graphical abstract

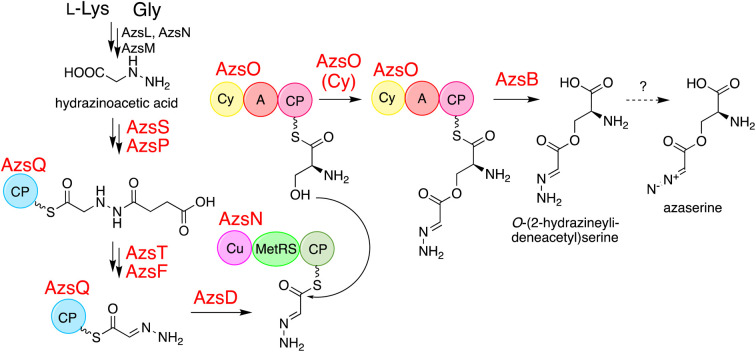

## Introduction

More than 200 natural products with nitrogen–nitrogen (N–N) bonds have been identified in nature.^1,2^ Many of these natural products possess useful biological activities because of their N–N bonds. Although the biosynthetic mechanisms of N–N bond formation are not fully understood, several studies have identified enzymes responsible for the biosynthesis of N–N bonds.^3,4^ For instance, Spb40 and its homologs have been reported to synthesize a hydrazine moiety from *N*^6^-hydroxylysine and an amino acid (*e.g.*, glycine and glutamate).^5–11^ In the biosynthesis of s56-p1, a dipeptide natural product with a unique hydrazone unit, lysine is oxidized to *N*^6^-hydroxylysine by the lysine *N*-hydroxylase Spb38. Furthermore, N–N bond formation is catalyzed by the didomain protein Spb40 composed of the N-terminal cupin domain and the C-terminal methionyl-tRNA synthetase (MetRS)-like domain. Finally, the oxidase Spb39 catalyzes the oxidation of the C–N bond followed by hydrolysis, resulting in hydrazinoacetic acid (HAA) synthesis.^7^ Furthermore, the heme-dependent enzyme KtzT has been reported to catalyze intramolecular cyclization of *N*^5^-hydroxyornithine to synthesize piperazic acid, a building block for nonribosomal peptides.^12^ The metalloenzyme SznF has been reported to synthesize *N*^δ^-hydroxy-*N*^ω^-methyl-*N*^ω^-nitroso-l-citrulline from *N*^ω^-methyl-l-arginine in streptozotocin biosynthesis.^13,14^ However, there are still many natural products with N–N bonds whose biosynthetic pathways remain enigmatic.

The diazo group is an N–N bond-containing functional group. It can be utilized as a tool for chemical probes and can confer bioactivity to compounds because of its high reactivity.^2,15,16^ Recently, the mechanisms of diazo group formation in the biosynthesis of several secondary metabolites (cremeomycin, alazopeptin, tasikamides, and avenalumic acid) from microorganisms has been elucidated.^17–21^ In these mechanisms, the distal nitrogen atom of the diazo group is derived from nitrous acid synthesized by the secondary metabolism-specific nitrous acid biosynthetic pathway, that is the aspartate/nitrosuccinate (ANS) pathway.^20,22^ The enzymes responsible for diazo group synthesis can be divided into two groups. CreM, Aha11, and AvaA6 in cremeomycin, tasikamides, and avenalumic acid biosynthesis, respectively, are ATP-dependent ligases that activate nitrous acid using ATP to synthesize the diazo group.^17,19,21^ In contrast, AzpL in alazopeptin biosynthesis is a putative membrane-bound protein, the reaction mechanism of which remains unclear.^18^ Although information on diazo group biosynthesis is accumulating rapidly, there are still several natural products, such as azaserine^23,24^ and thrazarine,^25,26^ whose diazo group biosynthesis remains unclear. Therefore, it is still unclear whether all diazo groups of secondary metabolites are biosynthesized using nitrous acid.

Azaserine, isolated from *Streptomyces fragilis* and *Glycomyces harbinensis*, is a non-proteinogenic amino acid possessing a diazo group. There are also several other compounds with similar structures, such as 6-diazo-5-oxonorleucine (DON) and thrazarine.^25–29^ Azaserine has antitumor and antibacterial activities due to its structural similarity to glutamine and is one of the most studied l-glutamine antagonists.^30^ Azaserine is an essential reagent used in basic research as it serves as an amidotransferase inhibitor to hinder hexosamine biosynthesis^31^ and as a tool for generating model rats with pancreatic cancer.^32^ Despite its useful features, the azaserine biosynthetic pathway remains to be elucidated.

Nonribosomal peptide synthetases (NRPSs) are a class of enzymes that are responsible for the biosynthesis of nonribosomal peptides.^33^ NRPSs are large multifunctional enzymes that generally consist of adenylation (A), condensation (C), and carrier protein (CP) domains. These domains play different roles in peptide bond synthesis. The A domain activates a substrate and covalently connects it to the phosphopantetheinyl moiety attached to the CP domain. The C domain catalyzes the condensation of CP domain bound amino acids (or peptides) to form a peptide bond. Some C domains catalyze different reactions, such as ester bond synthesis.^34,35^ In some cases, the C domain is substituted for the heterocyclization (Cy) domain, which catalyzes peptide bond synthesis and heterocyclization to synthesize an oxazoline ring (from Ser or Thr) or thiazoline ring (from Cys).^36,37^ Some Cy domains do not catalyze both reactions and catalyze only one of these reactions,^38–40^ indicating that understanding the function of Cy domain variants is important for predicting the products of orphan biosynthetic gene clusters.

**Fig. 1:**
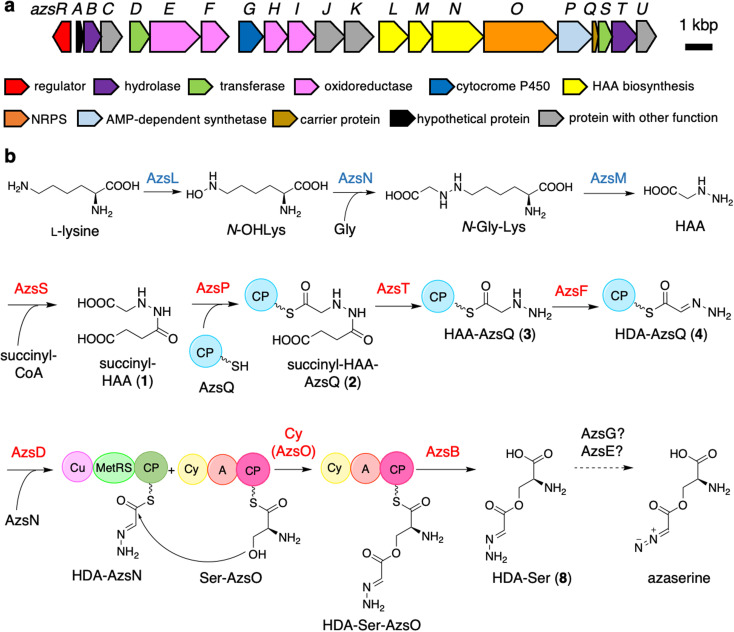
Biosynthetic gene cluster of azaserine in *Streptomyces fragilis* (a) and proposed azaserine biosynthetic pathway (b). A, adenylation; Cy, heterocyclization; CP, carrier protein; Cu, cupin; MetRS, methionyl-tRNA synthetase-like domain. The proteins whose functions were predicted according to the previously reported HAA biosynthesis are shown in blue. The proteins characterized *in vitro* are shown in red.

In this study, we identified the azaserine biosynthetic gene cluster and proposed an overall biosynthetic pathway based on *in vitro* analysis as well as the predicted functions of homologous enzymes (Fig. 1). Heterologous expression of the cluster in *Streptomyces albus* combined with the forced expression of azaserine biosynthetic gene *azsR*, which encodes a LuxR-family^41^ transcriptional regulator, resulted in the production of azaserine. Azaserine was synthesized from HAA, presumably *via* the oxidation of the hydrazine moiety, providing the first example of a nitrous acid-independent diazo group biosynthetic pathway. Meanwhile, AzsO, an NRPS consisting of Cy, A, and CP domains, was shown to catalyze ester bond formation, providing the first example of an NRPS Cy domain that catalyzes ester bond formation.^42–44^ This study provides important insights into the biosynthesis of non-proteinogenic amino acids and diazo compounds in microbial secondary metabolism.

## Results

### Genome-scanning and bioinformatics analysis of the putative azaserine biosynthetic gene cluster

Initially, we expected that the diazo group of azaserine would also be biosynthesized using nitrous acid biosynthesized by the ANS pathway.^20^ However, we could not find any genes related to the ANS pathway in the genome of *S. fragilis*, the azaserine producer whose genome sequence is available in the genome database (accession number: NZ_BEVZ01000003.1). Therefore, we assumed an alternative route for diazo group biosynthesis, in which HAA^7^ is the core intermediate, because the hydrazine moiety can be converted to the diazo group by two-step oxidation. By searching for a biosynthetic gene cluster containing HAA biosynthetic genes, we discovered a putative azaserine biosynthetic gene cluster consisting of 21 genes (Fig. 1a) in the genome of *S. fragilis*. We named this gene cluster as *azs* cluster. When we searched for homologous clusters in the genome database, we discovered approximately 50 putative azaserine biosynthetic gene clusters. These clusters were analyzed and classified using antiSMASH and BiGSCAPE (Fig. S1†).^45,46^

The *azs* cluster encodes the enzymes AzsL, AzsM, and AzsN, which show amino acid sequence similarities to Sbp38, 39, and 40, respectively, which are the enzymes responsible for HAA biosynthesis in s56-p1 biosynthesis.^7^ AzsN has an extra carrier protein domain (see the schematic representation of AzsN in Fig. 1b), which does not exist in Sbp40. In addition, the *azs* cluster encodes *N*-acetyltransferase (AzsS), AMP-dependent synthetase (AzsP), CP (AzsQ), peptidase (AzsT), dehydrogenase (AzsF), and 3-oxoacyl-acyl carrier protein (ACP) synthase (AzsD). These proteins, including AzsL, AzsM, and AzsN, show amino acid sequence similarities to proteins involved in the biosynthesis of triacsin (Table 1).^8,9^ Del Rio Flores *et al.* showed that Tri31, Tri29, and Tri22 are responsible for the synthesis of 2-hydrazineylideneacetyl (HDA)-CP (Tri30) by the following sequential reactions: succinylation of HAA (by Tri31), loading of succinyl-HAA to CP (by Tri29), and hydrolysis of the succinyl moiety accompanied with dehydrogenation (by Tri22).^9^ Because the HDA moiety is a suitable precursor for the diazoacetyl moiety of azaserine, similar reactions should be involved in the biosynthesis of azaserine. Because Tri13, the homolog of AzsD, was shown to catalyze the transfer of the HDA moiety between two carrier proteins (Tri30 to Tri20),^9^ AzsD was deduced to have a similar function. AzsD may catalyze the transfer of the acyl moiety between two CPs, AzsQ and the C-terminal CP domain of AzsN. Tri14, the homolog of AzsT, was predicted to catalyze the cleavage of the extended product synthesized by a type II polyketide synthase (PKS).^9^ Since *azs* clusters do not contain PKS genes, the function of AzsT was unclear.

**Table 1:** Predicted functions of *azs* gene products

Gene	Accession number	Predicted function	Protein homolog (related metabolite)	Identity [%]
*azsR*	WP_108953661.1	LuxR family regulator	AGG35693.1 (arginomycin)	41.0
*azsA*	WP_108953662.1	Hypothetical protein		
*azsB*	WP_108953663.1	Thioesterase	ScoT (coelimycin P1)	39.0
			Spb9 (s56-p1)	34.5
*azsC*	WP_108953664.1	Isocitrate lyase	LedA (le-pyrrolopyrazines)	36.0
*azsD*	WP_108954055.1	3-Oxoacyl-ACP synthase	Tri13 (triacsin)	52.6
*azsE*	WP_108953665.1	Iron-containing redox enzyme		36.0
*azsF*	WP_108953666.1	Acyl-CoA dehydrogenase	Tri22 (triacsin)	65.9
*azsG*	WP_108954056.1	Cytochrome P450	AAL06684.1 (C-1027)	36.0
			CAJ88189.1 (stambomycin)	24.7
*azsH*	WP_108953667.1	Saccharopine dehydrogenase	Tri23 (triacsin)	57.0
*azsI*	WP_108953668.1	Saccharopine dehydrogenase	Tri24 (triacsin)	68.0
*azsJ*	WP_108953669.1	ATP-grasp protein		
*azsK*	WP_108953670.1	MFS transporter		
*azsL*	WP_108953671.1	Monooxygenase	Tri26 (triacsin)	69.0
*azsM*	WP_108953672.1	FAD-binding oxidoreductase	Tri27 (triacsin)	61.8
*azsN*	WP_108953673.1	Cupin/class I tRNA ligase/carrier protein	Tri28 (triacsin)	72.9
*azsO*	WP_108953674.1	NRPS (Cy-A-CP)	CtaC (cystothiazole)	39.0
*azsP*	WP_108953675.1	AMP-binding protein	Tri29 (triacsin)	67.2
*azsQ*	WP_108953676.1	Carrier protein	Tri30 (triacsin)	66.0
*azsS*	WP_108953677.1	*N*-acetyltransferase	Tri31 (triacsin)	78.4
*azsT*	WP_108953678.1	Peptidase C45	Tri14 (triacsin)	56.0
*azsU*	WP_108953679.1	Phosphopantetheinyl transferase	Tri15 (triacsin)	45.0

AzsE is a putative redox enzyme, which was predicted to have a HemeO-like monooxygenase family domain at the C-terminus (528–706) using Pfam analysis.^47–49^ AzsE also shows a weak similarity (27% identity) to GvgC, which was shown to be involved in the biosynthesis of 4-formylaminooxyvinylglycine in *Pseudomonas fluorescens* WH6 by gene inactivation experiments.^50,51^ AzsG is a cytochrome P450 with a weak similarity (24.7% identity) to SAMR0479 in stambomycin A biosynthesis in *Streptomyces ambofaciens* ATCC23877.^52^ SAMR0479 was shown to catalyze C–H oxidation to produce a hydroxy group.^53^ Either of the two putative monooxygenases, AzsE and AzsG, may be involved in the oxidation of the HDA moiety to synthesize a diazo group.

AzsO is an NRPS composed of Cy, A, and CP domains. The A domain of AzsO was predicted to recognize cysteine using antiSMASH.^45^ However, azaserine does not contain cysteine. Therefore, this enzyme should recognize serine. Usually, a Cy domain catalyzes two reactions, peptide bond formation and heterocyclization, to synthesize oxazoline or thiazoline ring from serine (threonine) or cysteine, respectively.^36,37^ Comparison of the sequence of AzsO with several representative Cy domains indicated that AzsO has a DxxxxD motif, which is conserved among Cy domains and is known to be important for peptide bond formation.^54^ However, the conserved Thr and Asp residues, which were shown to be important for the heterocyclization reaction, were replaced by Asn in AzsO (Fig. S2†).^36,37^ All AzsO homologs discovered from the genome database have above-mentioned Thr to Asn substitutions (Fig. S2†). In contrast, the Asp residues are retained in some homologs. Because of the substitution in two catalytic residues, we speculated that the Cy domain of AzsO should have a different function from that of a typical Cy domain and catalyze ester bond synthesis according to the structure of azaserine. In addition, structural prediction of AzsO using ColabFold^55,56^ suggested that it has a docking domain at the N-terminal portion of the Cy domain, indicating that AzsO presumably interacts with a *trans*-CP (Fig. S3†). Because AzsO lacks a thioesterase domain, it was expected that the putative thioesterase AzsB would cleave the product from the CP domain of AzsO.

AzsH and AzsI are putative saccharopine dehydrogenases that catalyze the hydrolysis of saccharopine to l-lysine and α-ketoglutaric acid. AzsH and AzsI show similarities to Tri23 and Tri24, respectively, encoded by the triacsin biosynthetic gene cluster.^8^ These enzymes are apparently not crucial for azaserine biosynthesis because disruption of *tri23* and *tri24* genes did not influence the production of triacsin.^8^ Therefore, these enzymes are probably important for the supply of a precursor, l-lysine, to enhance the yield of the final product. We also predicted that AzsC, which is a homolog of an isocitrate lyase in the glyoxylate cycle, would be important for the supply of another precursor, succinate. AzsR, a LuxR family transcriptional regulator,^57,58^ is expected to control the expression of *azs* genes, because some cluster-situated LuxR family regulators are known to activate related secondary metabolite biosynthetic genes. AzsK is a putative transporter, which may be related to azaserine secretion and/or resistance. AzsA is a hypothetical protein, and AzsJ is an ATP-grasp protein. We speculated that these proteins may not be involved in the biosynthesis of azaserine because these protein homologs are not encoded by some putative azaserine biosynthetic gene clusters (Fig. S1†).

### Heterologous expression of the *azs* cluster in *Streptomyces albus*

**Fig. 2:**
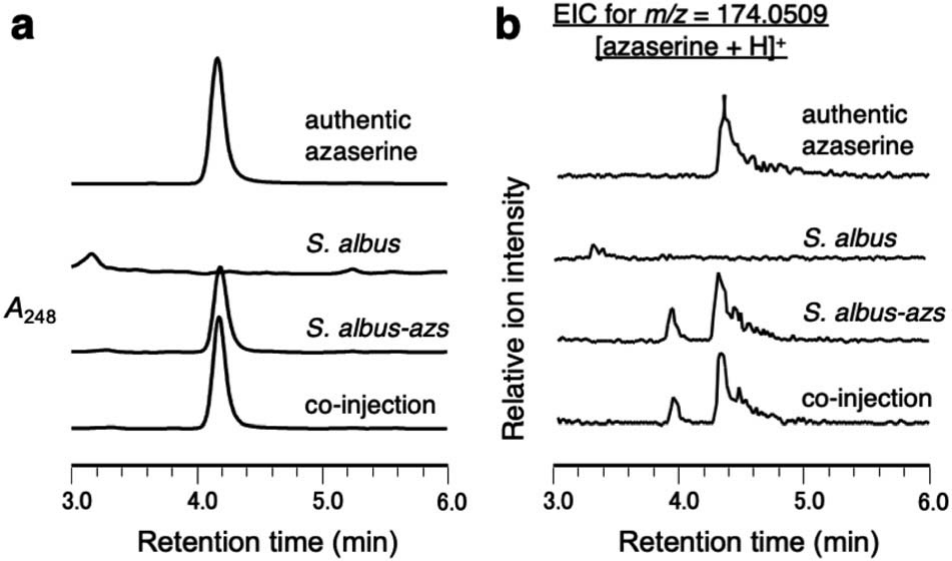
Heterologous expression of the *azs* cluster in *S. albus*. (a) LC analysis of metabolites produced by *S. albus-azs*. UV chromatograms extracted at 248 nm. (b) LC-HRMS analysis of metabolites produced by *S. albus-azs*. Extracted ion chromatograms of *m*/*z* 174.0509, corresponding to [M + H]^+^ ion of azaserine.

First, we confirmed by heterologous expression that the putative azaserine biosynthetic gene cluster was responsible for the production of azaserine. pTYM19-*azs* harboring the whole *azs* cluster (from *azsR* to *azsU*; Fig. 1a) and pHKO4-*azsR* carrying *azsR* under the control of *tipA* promoter were introduced into *Streptomyces albus* J1074, resulting in *S. albus-azs.* This strain possesses two copies of *azsR*; one is under the control of *tipA* promoter and the other has its own native promoter. Then, we analyzed the metabolic profile of the recombinant strain using liquid chromatography-high resolution mass spectrometry (LC-HRMS) (Fig. 2a and S4†). A compound with the same *m*/*z* ([M + H]^+^ = C_5_H_8_N_3_O_4_^+^, *m*/*z* obs. 174.0508, calcd 174.0509), ultraviolet (UV) absorption spectrum (*λ*_max_ at 248 nm), MS/MS fragments, and retention time as commercially available azaserine was observed (Fig. 2 and S4†). This compound was not observed in the strain without *azs* cluster. Co-injection of the compound produced by the *S. albus-azs* with the authentic azaserine resulted in no additional peak, confirming that these compounds are the same (Fig. 2). These results demonstrate that this cluster is responsible for azaserine biosynthesis.

### *In vitro* analysis of HAA succinylation and loading of succinyl-HAA onto CP

**Fig. 3:**
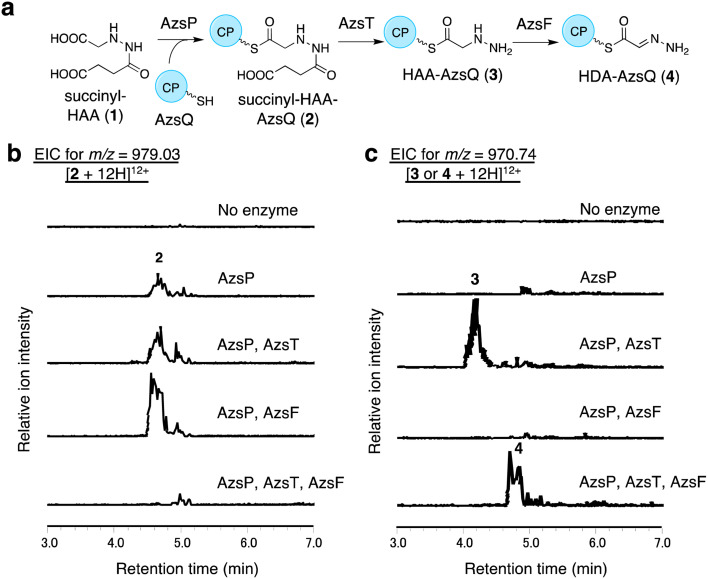
*In vitro* analysis of AzsP, AzsT, and AzsF. (a) Overview of the pathway catalyzed by these enzymes. (b and c) *In vitro* analysis of AzsP, AzsT, and AzsF. (b) Extracted ion chromatograms of *m*/*z* 979.03, corresponding to [M + 12H]^12+^ ion of 2. (c) Extracted ion chromatograms of *m*/*z* 970.74, corresponding to [M + 12H]^12+^ ion of 3. All reactions contained synthetic 1, *holo*-AzsQ, and FAD. Because the molecular weights of 3 and 4 differ only by 2 Da, both compounds could be observed in the chromatograms. However, it should be noted that 3 and 4 were detected at different retention times. At least three independent replicates were performed for each assay. All the results showed the same trend.

We performed *in vitro* analysis of enzymes that were predicted to be involved in the reactions after HAA synthesis using recombinant AzsB, D, F, O, P, S, T, Q, and N proteins (see Materials and methods for details of the preparation of recombinant proteins; Fig. S5†). We prepared an AzsT homolog from *Streptomyces niger* (Sn_AzsT) because AzsT from *S. fragilis* could not be obtained as a soluble protein (Fig. S1 and S5†). The reaction cascade starting from HAA should be similar to that of triacsin biosynthesis: succinylation of HAA catalyzed by AzsS (Tri31), loading of succinyl-HAA (1) to AzsQ (CP, Tri30) by AzsP (Tri29), and hydrolysis of the succinyl moiety followed by oxidation catalyzed by AzsF (Tri22). First, we analyzed the activity of AzsS using chemically synthesized HAA.^59^ When AzsS was incubated with HAA and succinyl-CoA, production of succinyl-HAA (1) was observed using LC-MS analysis equipped with a HILIC column (Fig. S6†). Next, we examined the functions of AzsP and AzsQ. When AzsP was incubated with *holo*-AzsQ and synthetic 1, succinyl-HAA-AzsQ (2) was observed by LC-HRMS (Fig. 3b). The binding of the succinyl-HAA moiety was further confirmed by the phosphopantetheine ejection assay (Fig. S7†).^60^

### AzsT and AzsF catalyze HDA-AzsQ (4) synthesis from succinyl-HAA-AzsQ (2)

We analyzed the function of AzsF, a homolog of Tri22. Tri22 was reported to catalyze two reactions: hydrolysis of the succinyl moiety of succinyl-HAA-CP and subsequent dehydrogenation to synthesize HDA-CP.^9^ Contrary to our expectation, incubation of AzsF with enzymatically synthesized 2 did not result in 4. Therefore, we assumed that an additional hydrolase is required for the conversion of 2 to 4. We speculated that AzsT, a C45 family peptidase, is likely responsible for the hydrolysis of the succinyl group. When AzsT was incubated with enzymatically synthesized 2, the formation of HAA-AzsQ (3) was observed using LC-HRMS (Fig. 3c). When AzsF was added to the reaction mixture, a new compound with a molecular weight similar to that of 3 but with a different retention time, was detected (Fig. 3c). We predicted that this compound would be HDA-AzsQ (4), which is different from 3 by 2 Da. To confirm this, the phosphopantetheine ejection assay was performed (Fig. S7†). The *m*/*z* values of fragments derived from 3 and 4 were consistent with their predicted structures (Fig. S7†). These results demonstrate that AzsT and AzsF catalyze the hydrolysis of 2 and oxidation of 3, respectively, to synthesize 4.

### AzsD catalyzes the transfer of the HDA moiety of AzsQ onto the C-terminal CP-domain of AzsN

**Fig. 4:**
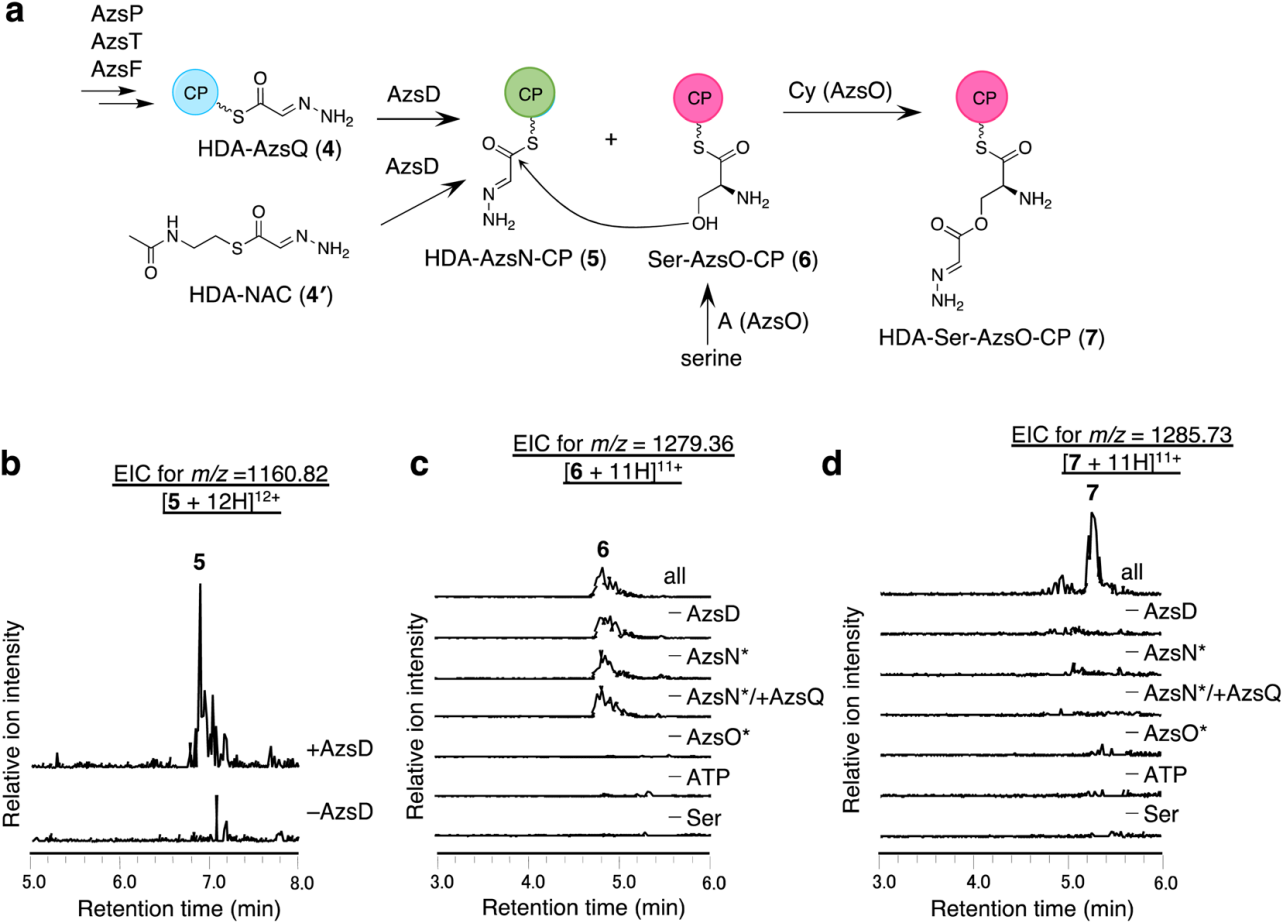
*In vitro* analysis of AzsD, AzsN, and AzsO. (a) The reactions catalyzed by these enzymes. (b) *In vitro* analysis of AzsD. Extracted ion chromatograms of *m*/*z* 1160.82, corresponding to [M + 12H]^12+^ ion of 5. (c and d) *In vitro* analysis of AzsO and AzsB. (c) Extracted ion chromatograms of *m*/*z* 1279.36, corresponding to [M + 11H]^11+^ of 6. (d) Extracted ion chromatograms of *m*/*z* 1285.73, corresponding to [M + 11H]^11+^ of 7. Both full-length and truncated proteins were used for AzsN and AzsO in this reaction. The full-length proteins are marked with * in (c) and (d).

We analyzed the function of AzsD, which belongs to the 3-oxoacyl-ACP synthase III family. AzsD was expected to catalyze the transfer of the HDA moiety of AzsQ to another CP, presumably, the C-terminal CP domain of AzsN, analogous to Tri13.^9^ For analyzing the function of AzsD, the CP domain of AzsN (AzsN-CP) was prepared as a truncated protein (Fig. S5†). Incubation of AzsD with *holo*-AzsN-CP and enzymatically prepared HDA-AzsQ (4) resulted in the synthesis of HDA-AzsN-CP (5) (Fig. 4b). This result indicates that AzsD catalyzes the expected reaction. We also analyzed the function of AzsD using HDA-*N*-acetylcysteamine thioester (HDA-NAC; 4′), an analog of HDA-AzsQ. Incubation of 4′ and *holo*-AzsN-CP resulted in the synthesis of HDA-AzsN-CP (5), indicating that the transfer of the HDA moiety occurred nonenzymatically (Fig. S8a†). The addition of AzsD to the reaction mixture slightly stimulated HDA-AzsN-CP (5) production (Fig. S8a†). The nonenzymatic reaction did not proceed when the concentration of 4′ was reduced to 100 μM and HDA-AzsN-CP (5) was detected only in the presence of AzsD (Fig. S8a†). This result indicates that AzsD can accept HDA-NAC as a substrate. In addition, AzsD also catalyzed the transfer of the HDA moiety of 4′ to AzsQ (Fig. S8b†). These results showed that AzsD catalyzes the transfer of the HDA moiety between AzsQ and AzsN-CP.

### AzsO and AzsB synthesize *O*-(2-hydrazineylideneacetyl)serine (HDA-Ser)

We analyzed the function of AzsO, an NRPS with Cy-A-CP domains. AzsO was expected to catalyze ester bond synthesis between HDA and l-serine, according to the structure of azaserine. To enable LC-MS analysis of the reaction products attached to the CP domain of AzsO, we prepared the truncated CP domain of AzsO (AzsO-CP) (Fig. S5†). When *holo*-AzsO was incubated with *holo*-AzsN, HDA-NAC (4′), AzsD, l-serine, and *holo*-AzsO-CP, Ser-AzsO-CP (6) and HDA-Ser-AzsO-CP (7) were detected using LC-HRMS (Fig. 4c and d). The binding of l-serine and HDA-Ser was further confirmed by the phosphopantetheine ejection assay (Fig. S9†). Removal of either AzsO, ATP, or l-serine from the reaction mixture abolished the production of both compounds. In contrast, removal of *holo*-AzsN abolished only the production of HDA-Ser-AzsO-CP (7). In addition, when *holo*-AzsQ was included in the reaction mixture instead of *holo*-AzsN, the production of HDA-Ser-AzsO-CP (7) was also not observed. These results indicated that the Cy domain of AzsO can only use the HDA moiety attached to the *holo*-AzsN and cannot use 4′ or HDA moiety attached to the *holo*-AzsQ as a substrate. Finally, the removal of AzsD abolished the yield of 7, consistent with a previous observation that the transfer of the HDA moiety from 4′ to AzsN occurred by AzsD (Fig. S8†). These results indicate that AzsO (most likely its Cy domain) catalyzes the synthesis of the ester bond of HDA-Ser (8). In this reaction, the HDA-AzsO-CP was observed in the absence of AzsO, ATP, or l-serine, indicating that AzsD can also transfer the HDA moiety from HDA-NAC to AzsO-CP only when the A domain of AzsO cannot load l-serine onto the CP domain of AzsO (Fig. S10†). The data also indicate that AzsD has a promiscuous selectivity against CPs.

Next, we searched for the enzyme responsible for releasing HDA-Ser (8) from HDA-Ser-AzsO or HDA-Ser-AzsO-CP (7). The thioesterase AzsB appeared to be the most probable candidate for catalyzing this reaction. First, we analyzed the effect of AzsB on the acylated *holo*-AzsO-CP. The addition of AzsB to the reaction mixture abolished both Ser-AzsO-CP (6) and HDA-Ser-AzsO-CP (7), indicating that AzsB hydrolyzed these *holo*-AzsO-CP-bound compounds (Fig. S11a and b†). Next, we attempted to detect the product released from *holo*-AzsO-CP. However, we could not detect any detectable amount, presumably because of the low yield. We assumed that the low yield could be ascribed to the weak interaction between the truncated *holo*-AzsO-CP and Cy domain of AzsO. In addition, the instability of truncated CP proteins (*holo*-AzsO-CP) seemed to affect the yield. Therefore, only the full-length *holo*-AzsO protein was used for the *in vitro* assay. The addition of AzsB to the reaction mixture (containing *holo*-AzsN, HDA-NAC (4′), AzsD, *holo*-AzsO, and l-serine) resulted in the production of a compound with the same retention time as authentic HDA-Ser (8) (Fig. S11†), which was synthesized by the reduction of azaserine using sodium borohydride (Fig. S12†). These results indicate that AzsB catalyzes the hydrolysis of HDA-Ser-AzsO to cleave HDA-Ser (8) from AzsO.

### Site-directed mutagenesis of the Cy domain of AzsO

To obtain further insight into the mechanism of ester bond formation catalyzed by AzsO, site-directed mutagenesis of the Cy domain of AzsO was performed. As described above, the DxxxxD motif, previously reported to play an important role in peptide bond formation, is highly conserved among AzsO homologs (Fig. S2†).^36^ In contrast, both conserved Thr and Asp residues, which have been reported to be important for heterocyclization, are replaced by Asn (Asn414 and Asn447, respectively) in AzsO.^36,37^ To examine the importance of these residues in ester bond synthesis, AzsO variants (D193A, D198A, N414A, N414T, N447A, and N447D) were prepared (Fig. S13a†). *In vitro* analysis using *holo*-AzsO (wild-type or each variant), *holo*-AzsN-CP, HDA-NAC (4′), AzsD, l-serine, and *holo*-AzsO-CP showed that these variants did not catalyze ester bond formation because HDA-Ser-AzsO-CP (7) was not detected in the reaction of any of these variants (Fig. S13c†). However, each AzsO variant was shown to be capable of activating and binding l-serine because Ser-AzsO-CP (6) was detected (Fig. S13b†). In addition, none of these variants synthesized a compound that has *m*/*z* corresponding to cyclodehydrated HDA-Ser-AzsO-CP (7′) (Fig. S13d†). These results confirmed that each amino acid replacement in the Cy domain did not affect the function of the A and CP domains of each AzsO variant. Taken together, these results show that the four residues (D193, D198, N414, and N447) of the Cy domain of AzsO are important for ester bond synthesis.

## Discussion

In this study, we identified most of the azaserine biosynthetic pathway (Fig. 1b). According to the previous studies, AzsL, AzsM, and AzsN were assumed to synthesize HAA from l-lysine and glycine.^7,9^ Our results indicated that HAA is used for azaserine synthesis as follows. HAA is succinylated to succinyl-HAA (1) by AzsS. AzsP activates succinyl-HAA (1) and loads it onto AzsQ, resulting in succinyl-HAA-AzsQ (2). AzsT catalyzes the hydrolysis of the succinyl group of succinyl-HAA-AzsQ (2) to produce HAA-AzsQ (3). AzsF synthesizes HDA-AzsQ (4) from HAA-AzsQ (3) *via* dehydrogenation. AzsD transfers the HDA moiety from AzsQ to the CP domain of AzsN to synthesize HDA-AzsN (4). AzsO catalyzes the condensation of the HDA moiety with the hydroxy group of l-serine attached to the CP domain of AzsO to synthesize HDA-Ser-AzsO. AzsB releases HDA-Ser (8) *via* hydrolysis. HDA-Ser (8) is presumably oxidized by one of the oxidoreductases in the cluster, such as the putative cytochrome P450 AzsG, resulting in azaserine.

It is interesting that AzsN is a bifunctional enzyme; the CP domain of AzsN is used as a carrier of the HDA moiety in the late stage of the azaserine biosynthetic pathway, while the other domains (cupin and MetRS) participate in HAA synthesis in the early stage. Upon searching for AzsN homologs with a CP domain, we observed that most of them are encoded within the putative azaserine biosynthetic gene cluster (Fig. S1†). Furthermore, we noted that, all AzsN homologs with a CP domain are encoded just upstream of each *azsO* homolog. Therefore, we hypothesize that the CP domain may have initially been encoded upstream of *azsO* as an independent gene in the ancestral *azs* cluster. Subsequently, during the evolution of the azaserine biosynthetic gene cluster, gene fusion likely occurred between the CP domain-encoding gene and *azsN*, which originally encoded cupin and MetRS domains.

Although a series of reactions to produce HDA-AzsQ (4) in azaserine biosynthesis are similar to those in triacsin biosynthesis, there is a difference: conversion of succinyl-HAA-CP to HDA-CP requires two enzymes, AzsT and AzsF, in azaserine biosynthesis, whereas only Tri22 is required in triacsin biosynthesis.^9^ AzsT is responsible for desuccinylation, although it shows amino acid sequence similarity to the peptidase Tri14 (56% identity), which is responsible for the release of the final product synthesized by the polyene-synthesizing type II polyketide synthase from ACP in triacsin biosynthesis. In addition, AzsF catalyzed dehydrogenation only, although it shows amino acid sequence similarity to the dehydrogenase Tri22 (66% identity), which has been shown to catalyze desuccinylation and dehydrogenation by *in vitro* analysis. During the peer review of this manuscript, Van Cura *et al.* reported the biosynthesis of azaserine.^61^ They also insisted that AzsF (named as AzaM) was found to catalyze both reactions.^61^ In their study, *in vitro* analysis of AzsS, Q, P, and F (named as AzaB, AzaQ, AzaC, and AzaM, respectively) showed that these four enzymes were sufficient for HDA-AzsQ synthesis, indicating that AzsF should catalyze both desuccinylation and dehydrogenation. In their study, however, different conditions were used (higher concentrations of the enzymes and slightly higher pH), and therefore we speculate that some peptidases which could not be completely removed by the Ni^2+^ affinity chromatography hydrolyzed the amide bond of the succinyl-HAA moiety or that nonenzymatic hydrolysis occurred. Further analysis is required to understand the reason for this difference.

AzsO is an unusual NRPS with a Cy domain that catalyzes ester bond formation between l-serine and HDA. Although there are several examples of C domains catalyzing ester bond formation and the Cy domain is closely related to the C domain,^33,35,42–44,62^ no Cy domain catalyzing this reaction has been reported.^34,63^ To our knowledge, AzsO is the first example of an NRPS with a Cy domain that targets a β-hydroxyl group to form an ester bond. Two amino acid residues (Thr and Asp) reported to be important for heterocyclization were found to be not completely conserved among AzsO homologs (Fig. S2†). Mutation analysis of these two residues indicated that they are important for ester bond formation. In particular, replacement of the conserved Thr could be an important signature to distinguish between the canonical Cy and ester bond-synthesizing Cy domains, considering the sequence comparison of AzsO homologs (Fig. S2†). A BLAST search using the Cy domain of AzsO as a query resulted in the discovery of 129 Cy domains with substitutions of the conserved Thr for Asn (among 1165 putative Cy domains), indicating that ester bond-synthesizing Cy domains could be widely used in secondary metabolism (Fig. S14†). However, the mechanism by which AzsO catalyzes *O*-acylation of the β-hydroxy group remains unclear. Further understanding of the catalytic mechanisms of such Cy domains is important to improve the accuracy of the bioinformatics tools used for predicting the structures of secondary metabolites from genome information.

Despite the structural elucidation of some Cy domains, the mechanism by which the peptide bond is synthesized remains unclear, although the heterocyclization reaction catalyzed by the Cy domain is well understood (Fig. S15†).^36,37,40,64,65^ It has been proposed that structural changes are required for the Cy domain to catalyze heterocyclization after peptide bond formation.^64,65^ This feature may hinder the prediction of the mechanism for the peptide bond synthesis reaction. Nonetheless, it is generally believed that the peptide bond is synthesized through the direct condensation of alpha amine with thioester (Fig. S15a†). Recently, Shi *et al.* proposed that the Cy domain from photoxenobactin biosynthesis catalyzes peptide bond formation *via* thioester bond synthesis, followed by the immediate transfer of the acyl moiety to synthesize peptide bond^66^ in analogy with native chemical ligation^67^ (Fig. S15b†). It is an interesting coincidence that the Cy domain of AzsO catalyzes ester bond formation using l-serine (Fig. S15b and c†) because AzsO seems to have a Cy domain that lacks acyl transfer activity. Therefore, the discovery of AzsO provides important insights into the reaction mechanism of peptide bond formation of Cy domains. Further analysis of these domains could lead to a better understanding of the evolution and diversity of enzymes belonging to C domain superfamily.^34^

*azs* cluster is the seventh biosynthetic gene cluster to be identified which produces diazo-containing compounds either as final products or intermediates; the biosynthetic gene clusters of cremeomycin,^20^ alazopeptin,^18^ tasikamide,^19^ avenalumic acid,^17^ kinamycin,^68^ and lomaiviticin^69,70^ were reported. Interestingly, the biosynthesis of the diazo moiety of azaserine differed from the previously reported pathways. Although we could not identify the enzymes responsible for diazo group biosynthesis in this study, *in vitro* reconstitution of HDA-Ser (8) biosynthesis strongly indicate that the diazo group is biosynthesized by the stepwise oxidation of HAA *via* HDA moiety synthesis. To date, all diazo group biosynthetic enzymes identified utilize the condensation of amines with nitrous acid.^17–21,68^ Therefore, the diazo group of azaserine is the first example of a diazo-containing natural product that is biosynthesized independently of nitrous acid. Thus, this study expands our knowledge of the diversity of the N–N bond biosynthetic machinery. We speculate that one of the putative oxidoreductases in the cluster, AzsE and AzsG, is responsible for the oxidation of the HDA moiety. However, despite our extensive attempts to identify these enzymes *in vitro* using recombinant enzymes to detect the oxidation of HDA-Ser (8) under different conditions (*e.g.*, buffer condition, different redox partners [putidaredoxin, phenazine methosulfate, and ascorbic acid], *etc.*), we could not observe this reaction. These enzymes may require a specific redox partner which is not easily available. Although *in vitro* reconstitution of HDA-Ser (8) biosynthesis strongly indicated that the diazo group synthesis is the last step of azaserine biosynthesis, we cannot completely exclude the possibility that the oxidation of the HDA moiety to the diazoacetyl moiety occurs at an earlier stage (*e.g.*, on the CP domain of AzsN, AzsO, or AzsQ) because we could not detect HDA-Ser (8) oxidation *in vitro*. Further *in vitro* analysis of these putative oxidoreductases is needed to elucidate the diazo group biosynthesis machinery in this pathway.

Through genome database analysis, we identified approximately 50 putative biosynthetic gene clusters that are likely to be involved in the biosynthesis of azaserine or its derivatives. Although only a few strains (*e.g.*, *Glycomyces harbinensis* and *Streptomyces fragilis*) have been reported to produce azaserine,^24^ these results indicate that azaserine or its derivatives may be produced by a wide variety of species. In addition, one of these clusters may also be responsible for the biosynthesis of thrazarine, an analog of azaserine, which is expected to be derived from threonine instead of glycine.^25,26^ Most of these clusters encode homologs of AzsL, M, and N (HAA synthesis); AzsS, T, F, and Q (HDA moiety synthesis); AzsO and B (ester bond synthesis); AzsG (cytochrome P450); and AzsE (iron-containing redox enzyme), indicating that these genes are a minimal set required for the biosynthesis of azaserine.

DON is a diazo-containing amino acid with a structure similar to that of azaserine. Moreover, it also has antitumor activity.^71,72^ The only difference between azaserine and DON is the atom at position 4; azaserine and DON have oxygen and carbon atoms at position 4, resulting in ester and ketomethylene moieties, respectively. Although they have similar structures, the diazo group of DON is synthesized *via* the condensation of nitrous acid with an amino group,^18^ while that of azaserine is synthesized *via* the oxidation of HAA. This observation provides a new example of how similar structures can be biosynthesized by completely different pathways, and it reemphasizes the remarkable diversity of secondary metabolism in microorganisms.

## Conclusions

In this study, we identified a large part of the azaserine biosynthesis pathway using *in vitro* analysis. This pathway includes a sequential enzymatic reaction cascade on multiple CPs, using HAA as a precursor to synthesize HDA-Ser. It is a nitrous acid-independent diazo group biosynthetic pathway that exploits stepwise oxidation of the HAA moiety. In addition, this pathway utilizes an unprecedented ester-bond-synthesizing Cy domain. These findings have deepened our knowledge of secondary metabolism in actinomycetes. Our study also provides useful information for improving bioinformatics pipelines for genome mining and enzyme engineering to generate “unnatural” natural products.

## Data availability

Experimental procedures and data are available in the ESI.†

## Author contributions

Y. S. and S. K. performed the biochemical experiments. Y. S., Y. K. and S. K. performed the bioinformatics analysis. The manuscript was written through contributions of all authors. Y. K., S. K., Y. S. and Y. O. design the research. All authors have given approval to the final version of the manuscript.

## Conflicts of interest

The authors declare no conflict of interest.

## Supplementary Material

SC-014-D3SC01906C-s001
