# Supplementary material for: In vitro characterization of nonribosomal peptide synthetase-dependent O-(2-hydrazineylideneacetyl)serine synthesis indicates a stepwise oxidation strategy to generate the α-diazo ester moiety of azaserine
Source: Chem Sci. 2023 Jul 3;14(33):8766–76. doi: 10.1039/d3sc01906c (PMC10445470; doi:10.1039/d3sc01906c)
Supplement: SC-014-D3SC01906C-s001 [file SC-014-D3SC01906C-s001.pdf]

Electronic Supplementary Material (ESI) for Chemical Science.
This journal is © The Royal Society of Chemistry 2023

***In vitro* characterization of nonribosomal peptide synthetase-dependent *O*-(2-hydrazineylideneacetyl)serine synthesis indicates the stepwise oxidation strategy to generate α-diazo ester moiety of azaserine**

Yusuke Shikai[a], Seiji Kawai[a], Yohei Katsuyama[a,b]*, Yasuo Ohnishi[a,b]

[a]Department of Biotechnology, Graduate School of Agricultural and Life Sciences, The University of Tokyo, 1-1-1 Yayoi, Bunkyo-ku, Tokyo 113-8657, Japan
[b]Collaborative Research Institute for Innovative Microbiology, The University of Tokyo, Bunkyo-ku, Tokyo 113-8657, Japan
*Correspondence: aykatsuhko@g.ecc.u-tokyo.ac.jp

Table of contents

Materials and Methods
Table S1. Primers used for cloning of the *azs* cluster.
Table S2. Primers used for the construction of plasmids for the preparation of Azs proteins.
Table S3. The final concentration of IPTG for gene expression.
Table S4. Primers used for site-directed mutagenesis of AzsO.
Figure S1. The *azs* cluster homologs discovered from the genome database.
Figure S2. Amino acid sequence alignment of the Cy domains of AzsO homologs and representative Cy domains (EpoB and BmdB).
Figure S3. The structure models of AzsO from *Streptomyces fragilis* constructed by AlphaFold2.
Figure S4. MS/MS spectra of authentic azaserine and the compound produced by *Streptomyces albus-azs*.
Figure S5. SDS-PAGE analysis of recombinant Azs proteins.
Figure S6. *In vitro* analysis of AzsS.
Figure S7. Phosphopantetheine ejection assay for the reaction products of AzsP, AzsT, and AzsF.
Figure S8. *In vitro* analysis of AzsD using HDA-NAC (**4′**) as a substrate.
Figure S9. Phosphopantetheine ejection assay for the reaction product of AzsO.
Figure S10. LC-HRMS analysis of HDA-AzsO-CP (**9**) in the reaction catalyzed by AzsD, AzsN, and AzsO.
Figure S11. LC-MS analysis of HDA-Ser (**8**) release catalyzed by AzsB.
Figure S12. Synthetic standard of HDA-Ser.
Figure S13. Site-directed mutagenesis of the Cy domain of AzsO.
Figure S14. Sequence logo around conserved motifs generated from 1,165 Cy domains.
Figure S15. Mechanism of the reactions catalyzed by Cy domains.
Figure S16. ^{1}H NMR data of Fmoc-HDA-NAC.

Materials and Methods

Materials

Streptomyces fragilis NBRC 12682 was obtained from the National Institute of Technology and Evaluation (NITE). *Streptomyces niger* JCM3158 was obtained from Japan Collection of Microorganisms (JCM). *Streptomyces albus* J1074 (*Streptomyces albidoflavus* J1074) used for the heterologous expression of *azs* genes was kindly provided from Prof. T. Kuzuyama. *Escherichia coli* DH5α (TaKaRa Bio Inc, Shiga, Japan) and *E. coli* HST08 (TaKaRa Bio Inc) were used for DNA manipulation, and *E. coli* BL21(DE3) (Merck KGaA, Darmstadt, Germany) was used for expressing recombinant proteins. In addition, *E. coli* S17-1 was used for conjugation. Enzymes used for DNA manipulation, including DNA polymerase and restriction enzymes, were purchased from TaKaRa Bio Inc. Primers were purchased from Thermo Fisher Scientific (Waltham, MA, USA). All chemicals used in this work were purchased from Sigma-Aldrich (St. Louis, MO, USA), New England BioLabs (Ipswich, MA, USA), and Tokyo Chemical Industry (Tokyo, Japan). Azaserine was purchased from Sigma-Aldrich.

Conjugational transfer

E. coli S17-1 harboring a plasmid for heterologous expression was inoculated into 5 mL of Luria-Bertani (LB) medium and incubated at 37°C overnight. The culture was transferred to 100 mL LB medium and incubated at 37°C until OD_{600} reached 0.6. The cells were harvested and washed twice with fresh LB medium and resuspended in 10 mL LB medium. Spores of *S. albus* J1074 in 100 µL of 10% glycerol were suspended in 0.5 mL TSB medium (30 g/L tryptic soy broth) and incubated at 50°C for 10 min. The spores and 500 µl of *E. coli* cells were mixed and inoculated on an MS agar plate (20 g/L soya flour, 20 g/L mannitol, and 20 g/L agar) containing 20 mM $MgCl_2$. After incubation at 30°C for 18-20 h, the plate was overlaid with an antibiotic solution. The antibiotic solution contained nalidixic acid (0.75 g/L), thiostrepton (0.75 g/L), and/or kanamycin (0.75 g/L). After incubation at 30°C for one week, several antibiotic-resistant colonies were obtained.

Construction of plasmids for heterologous expression

The primers used for plasmid construction are listed in **Table S1**. The whole *azs* cluster was divided into three fragments and amplified by PCR using appropriate primers (**Table S1**) and *S. fragilis* genomic DNA as a template. In addition, linearized

pTYM19 with the homologous region for gene cloning was amplified by PCR using appropriate primers (**Table S1**) and pTYM19 as a template.[1] The amplified DNA fragments were assembled by using In-Fusion (TaKaRa Bio Inc.), resulting in pTYM19-*azs*.

For the construction of pHKO4-*azsR*, *azsR* was amplified by PCR using appropriate primers (**Table S1**) and *S. fragilis* genomic DNA as a template and cloned into the NdeI and SphI sites of pHKO4 by using In-Fusion (TaKaRa Bio Inc.).

Heterologous expression of *azs* cluster in *S. albus* J1074-*azs*

S. albus J1074-*azs* was constructed by sequential transformation of *S. albus* J1074 using pTYM19-*azs* and pHKO4-*azsR*. *S. albus* J1074-*azs* was inoculated into 50 mL preculture medium (10 g/L glucose, 20 g/L dextrin, 5 g/L yeast extract, 5 g/L NZ-amine A, and 1 g/L $CaCO_3$) and cultured while shaking (150 rpm) at 30°C for 2 days. A portion (5 mL) of the preculture was inoculated into 100 mL ISP2 medium (4 g/L yeast extract, 10 g/L malt extract, and 4 g/L glucose) and cultured while shaking (150 rpm) at 30°C for 2 days. Then, 10 mg/L of thiostrepton was added to induce the expression of *azsR* and cultured while shaking (150 rpm) at 30°C for 1 day. A portion (10 mL) of the culture was harvested and stirred with 0.5 g of activated carbon powder (Nacalai Tesque, Kyoto, Japan) for 1 h. The mixture of cells and activated carbon powder were harvested by centrifugation at 13,100 g for 10 min. After the supernatant was removed, an equal volume of distilled water was added to the mixture, which was then gently resuspended. The sample was centrifuged at 13,100 g for 10 min, and the supernatant was removed. The compounds were extracted from the mixture using 10 mL of 60% methanol. After centrifugation, the supernatant was transferred to a new tube and evaporated to dryness under reduced pressure. The residual material was dissolved in 100 μL of 60% methanol.

The obtained samples were analyzed by liquid chromatography electrospray ionization mass spectrometry (LC-ESIMS) using the LC-2040C 3D Plus system (Shimadzu Corp., Kyoto, Japan) equipped with a COSMOSIL 2.5HILIC packed column (2.0 mm ID × 50 mm, Nacalai Tesque) coupled with a model LCMS-8040 liquid chromatograph tandem mass spectrometer (Shimadzu Corp.). The compounds were eluted with a linear gradient of acetonitrile/20 mM ammonium acetate. Then the UV chromatogram was obtained by photodiode array spectrophotometer in the LC-2040C 3D Plus system.

To obtain high resolution (HR) MS spectra, the samples were analyzed by LC-HRESIMS using the UFLC Nexera system (Shimadzu, Kyoto, Japan) equipped with a

COSMOSIL 2.5HILIC packed column (2.0 mm ID × 50 mm, Nacalai Tesque) coupled with the SCIEX Triple TOF 5600 system (SCIEX, Framingham, MA). The compounds were eluted with a linear gradient of acetonitrile/20 mM ammonium acetate.

Production and purification of recombinant AzsS, AzsP, AzsQ, AzsF, AzsD, AzsN, AzsN-CP, AzsO, AzsO-CP, AzsB, and Sn_AzsT

pColdI-*azsS*, pColdI-*azsP*, pColdI-*azsQ*, pColdI-*azsF*, pColdI-*azsD*, pColdI-*azsN*, pColdI-*azsN-CP*, pColdI-*azsO*, pColdI-*azsO-CP*, and pColdI-*azsB* were constructed by amplification of the genes with PCR using appropriate primers (**Table S2**) and genomic DNA of *S. fragilis* as a template followed by gene cloning using In-Fusion (TaKaRa Bio Inc.). pColdI-*Sn_azsT* was constructed by using the *azsT* gene amplified from the genomic DNA of *Streptomyces niger*. Each plasmid was introduced into *E. coli* BL21(DE3). To obtain *holo*-AzsQ, *holo*-AzsN, *holo*-AzsN-CP, *holo*-AzsO-CP, and *holo*-AzsO, corresponding plasmids were introduced to *E. coli* BL21(DE3) harboring pACYC-*sfp*.[2] To obtain Sn_AzsT, pColdI-*Sn_azsT* was introduced into *E. coli* with pTf-16 (TaKaRa Bio Inc.). Each obtained strain was cultured in 100 mL LB medium with ampicillin (1 mg/mL arabinose was added to LB medium for Sn_AzsT) while shaking (150 rpm) at 37°C until the OD_{600} reached 0.6. After cooling the culture on ice, isopropyl-D-thiogalactopyranoside (IPTG) was added to the culture to induce gene expression, and the culture was incubated while shaking (150 rpm) at 15°C for 24 h. The final concentration of IPTG is listed in **Table S3**. The cells were harvested by centrifugation and resuspended in lysis buffer (20 mM Tris-HCl, 10% glycerol, and 200 mM NaCl, pH 8.0). After lysis by sonication, the cell debris was removed by centrifugation. The recombinant protein was purified using His60 Ni Superflow Resin (TaKaRa Bio Inc.). The resin was added to the clear lysate and incubated with gentle shaking at 4°C for 1 h. After incubation, the resin was loaded onto a column and washed with 50 mL lysis buffer. The protein was eluted by lysis buffer containing different concentrations of imidazole. The buffer was desalted and concentrated with lysis buffer using an Amicon Ultra centrifugal filter with a suitable molecular mass cut-off (Merck Millipore, Burlington, MA, USA).

Chemical synthesis of hydrazine acetic acid (HAA)

HAA was synthesized according to the previously described procedure.[3] Bromoacetic acid (276 mg, 2 mmol) dissolved in ethanol was added to a three-necked flask equipped with a Dimroth condenser. The flask was substituted with nitrogen gas. Then, hydrazine (500 mg, 15 mmol) dissolved in ethanol was added dropwise. The

reaction mixture was refluxed at 80°C for 5 h. The reaction solution was then allowed to stand at 4°C overnight. The supernatant was obtained after centrifugation at 13,000 rpm for 30 min. The solution was evaporated to dryness under reduced pressure, resulting in HAA.

Chemical synthesis of *N*-succinyl-hydrazinoacetic acid (succinyl-HAA, 1)

9-Fluorenylmethyl carbazate (1 mmol) and succinic anhydride (1 mmol) were dissolved in 10 mL of methanol and the solution was stirred at room temperature for overnight. The reaction solution was evaporated to dryness under reduced pressure. The residual material was dissolved in 2 mL of dimethylformamide (DMF) with 500 μL of piperidine and the solution was stirred at room temperature for 2 h. Then, water and ethyl acetate were added to the solution. The aqueous layer was harvested and evaporated to dryness under reduced pressure. The residual material was dissolved in a mixture of 5 mL of water and 5 mL of acetonitrile. Bromoacetic acid (1 mmol) in acetonitrile was added dropwise to the solution and refluxed at 80°C for 4 h. The solution was evaporated to dryness, resulting in *N*-succinyl-hydrazinoacetic acid (succinyl-HAA) (**1**).

Chemical synthesis of *N*-acetylcysteamine thioester of 2-hydrazineylideneacetic acid (HDA-NAC)

Fmoc-HDA (1 mmol), WSC (1.1 mmol), DMAP (0.1 mmol), and NAC (1.1 mmol), were dissolved in dichloromethane and the solution was stirred at room temperature overnight under nitrogen atmosphere to obtain *N*-acetylcysteamine thioester of Fmoc-HDA (Fmoc-HDA-NAC). The liquid-liquid extraction was carried out with water and chloroform, and the chloroform layer was collected. The solution was evaporated to dryness under reduced pressure. Then, the residual material was applied to medium pressure liquid chromatography (MPLC) Purifi-Comp (Shoko Scientific) equipped with a silica gel column (Purif-Pack, Shoko Scientific). A linear gradient of hexane/ethyl acetate was used for the elution. Fmoc-HDA-NAC was dissolved in DMSO-d_6, and the structure was determined by the JNM-ECA500II NMR system (JEOL, Tokyo, Japan, **Figure S16**).

Fmoc-HDA-NAC. ^{1}H NMR (DMSO-d_6, 500 MHz): δ 8.05 (t, 1H, *J* = 5.5 Hz), 7.87 (d, 2H, *J* = 7.5 Hz), 7.724 (br, 2H), 7.50 (s, 1H), 7.39 (t, 2H, *J* = 7.5 Hz), 7.31 (t, 2H, *J* = 7.5 Hz), 4.53 (br, 2H), 4.29 (t, 1H, *J* = 7 Hz), 3.17 (q, 2H, *J* = 6.5 Hz), 2.93 (t, 2H, *J* = 6.5 Hz), 1.75 (s, 3H). LRMS (ESI) *m/z* $[M + H]^+$ calcd. for $C_{21}H_{22}N_3O_4S^+$ 412, found 412.

Since HDA-NAC was unstable, HDA-NAC was used for *in vitro* assay without further purification after deprotection of Fmoc-HDA-NAC as described below. Four milligram of Fmoc-HDA-NAC was dissolved in 100 µL of DMF with 5 µL of piperidine and the solution was stirred for 30 min at room temperature. After that, water (400 µL) and chloroform (400 µL) were added, and liquid-liquid extraction was then carried out. The aqueous layer was collected and used as 20 mM HDA-NAC.

***In vitro* analysis of AzsS**

A reaction mixture (100 µL) containing 20 µM AzsS, 10 mM HAA, 500 µM succinyl-CoA, and 200 mM Tris-HCl buffer (pH 7.5) was incubated at 30°C for 30 min. The reaction was quenched by adding methanol (100 µL). After centrifugation, the supernatant was analyzed by LC-ESIMS using the LC-2040C 3D Plus system (Shimadzu Corp.) equipped with a COSMOSIL 2.5HILIC packed column (2.0 mm ID × 50 mm, Nacalai Tesque) coupled with a model LCMS-8040 liquid chromatograph tandem mass spectrometer (Shimadzu Corp.). The compounds were eluted with a linear gradient of acetonitrile/20 mM ammonium acetate.

***In vitro* analysis of AzsP, AzsQ, AzsT, and AzsF**

A reaction mixture (100 µL) containing 5 µM AzsP, 50 µM AzsQ, 5 µM AzsT, 5 µM AzsF, 5 mM succinyl-HAA, 5 mM $MgCl_2$, 1 mM ATP, 100 µM FAD, and 200 mM Tris-HCl buffer (pH 7.5) was incubated at 30°C for 1 h. After centrifugation, the supernatant was analyzed by LC-HRESIMS using the UFLC Nexera system (Shimadzu) equipped with a BioResolve RP mAb packed column (2.1 mm ID × 100 mm, Waters, MA, USA) coupled with the SCIEX Triple TOF 5600 system (SCIEX). The compounds were eluted with a linear gradient of acetonitrile/water containing 0.1% formic acid. The phosphopantetheine ejection assay was carried out using this system.

***In vitro* analysis of AzsD and AzsN-CP**

A reaction mixture (100 µL) containing 5 µM AzsP, 50 µM AzsQ, 5 µM AzsT, 5 µM AzsF, 5 mM succinyl-HAA, 5 mM $MgCl_2$, 1 mM ATP, 100 µM FAD, and 200 mM Tris-HCl buffer (pH 7.5) was incubated at 30°C for 1 h. After incubation, 2 µM AzsD and 100 µM AzsN-CP were added, and the reaction mixture was further incubated at 30°C for 1 h. After centrifugation, the supernatant was analyzed by LC-HRESIMS following the same method used for AzsQ analysis.

***In vitro* analysis of AzsD using HDA-*N*-acetylcysteamine thioester (HDA-NAC; 4′)**

A reaction mixture (100 µL) containing 2 µM AzsD, 100 µM AzsN-CP or AzsQ, 1 mM or 100 µM HDA-NAC (**4′**) and 200 mM Tris-HCl buffer (pH 7.5) was incubated at 30°C for 30 min. After centrifugation, the supernatant was analyzed by LC-ESIMS using the LC-2040C 3D Plus system (Shimadzu Corp.) equipped with a BioResolve RP mAb packed column (2.1 mm ID × 100 mm, Waters) coupled with a model LCMS-8040 liquid chromatograph tandem mass spectrometer (LC-MS) (Shimadzu Corp.). The compounds were eluted with a linear gradient of acetonitrile/water containing 0.1% formic acid.

In vitro analysis of AzsO and AzsB

The intermediates binding to the CP domain of AzsO was detected as follows using the truncated *holo*-AzsO-CP. A reaction mixture (100 µL) containing 3 µM AzsD, 25 µM *holo*-AzsN, 50 µM *holo*-AzsO-CP, 2 µM AzsO, 100 µM HDA-NAC, 1 mM $MgCl_2$, 1 mM ATP, 1 mM L-Ser, and 100 mM potassium phosphate buffer (pH 8.0) was incubated at 30°C for 2 h. After centrifugation, the supernatant was analyzed by LC-HRESIMS following the same method used for AzsQ analysis.

A reaction mixture (100 µL) containing 3 µM AzsD, 0.2 µM AzsB, 2.5 µM *holo*-AzsN, 2 µM *holo*-AzsO, 400 µM HDA-NAC, 1 mM $MgCl_2$, 1 mM ATP, 1 mM L-Ser, and 100 mM potassium phosphate buffer (pH 8.0) was incubated at 30°C overnight. After centrifugation, the supernatant was analyzed by LC-ESIMS following the same method used for metabolite analysis to obtain the UV chromatogram.

Authentic HDA-Ser (**8**) was synthesized by reducing azaserine immediately before use. Azaserine (2 mg) and $NaBH_4$ (1.8 mg) were dissolved in 500 µL EtOH and incubated for 3 h. The synthesis of HDA-Ser was confirmed by HRMS and MS/MS (**Figure S12**) because it was highly unstable. The synthesized HDA-Ser (**8**) was purified by using MPLC (Purif-RP2, Shoko Scientific) equipped with a HILIC column (20 ID × 250 mm, Nacalai Tesque). The compound was eluted using linear gradient of acetonitrile/20 mM ammonium acetate. The fractions containing HDA-Ser were evaporated to dryness and dissolved in the reaction mixture without Azs proteins for analysis, because the retention time was affected and ionization efficiency decreased, by the compounds dissolved in the buffer.

Construction and analysis of AzsO variants

A DNA fragment with each mutation was amplified by PCR using an appropriate pair of primers listed in **Table S4** and pColdI-AzsO as a template. The obtained DNA fragment was cyclized using In-fusion and introduced into *E. coli* HST08. The

mutations were confirmed by DNA sequencing. AzsO variants were produced and purified as the same method used for wild-type AzsO.

In vitro reaction of the AzsO variants was performed using the method which was used for the detection of the intermediates binding to the CP domain of AzsO. The compounds were analyzed by LC-ESIMS using the LC-2040C 3D Plus system (Shimadzu Corp.) equipped with a BioResolve RP mAb packed column (2.1 mm ID × 100 mm, Waters) coupled with a model LCMS-8040 liquid chromatograph tandem mass spectrometer (Shimadzu Corp.). The compounds were eluted with a linear gradient of water/acetonitrile containing 0.1% formic acid.

Table S1. Primers used for cloning of the *azs* cluster.

Name	Sequence	Description
Sf-azs-F1	TGAACTGGGGCGAGCAACTG	
Sf-azs-R1	TGCGTCACCACCTGCTTCAG	
Sf-azs-F2	TGAAGCAGGTGGTGACGCAG	
Sf-azs-R2	AGGGTGAGGTCCACGTCGAA	
Sf-azs-F3	TTCGACGTGGACCTCACCCT	
Sf-azs-R3	CGACGGTAGAGGGTCCCGAT	
Sf-azs-Fpl	ATCGGGACCCTCTACCGTCGAAGCTTGGCGTAATCATGGT	HindIII site is underlined.
Sf-azs-Rpl	CAGTTGCTCGCCCCAGTTCATCTAGAGGATCCCCGGGTAC	XbaI site is underlined.
azsR_F	AAGGGAGCGGACATATGACGGAAGGCGCAGACAGG	NdeI site is underlined.
azsR_R	TCCTGCCCAAGCTTGCATGCTCAGACGACGATCTCGGTCT	SphI site is underlined.

Table S2. Primers used for the construction of plasmids for the preparation of Azs proteins.

Name	Sequence	Description
azsB_F	TCGAAGGTAGGCATATGAGCACCTCCCCCTGGTT	NdeI site is underlined.
azsB_R	CGACAAGCTTGAATTCTCAGACAACCTCCACATGCT	EcoRI site is underlined.
azsD_F	TCGAAGGTAGGCATATGTCCGGCCCCGCCTACGTC	NdeI site is underlined.
azsD_R	CGACAAGCTTGAATTCTCATCGTGACTCCCCGGTCT	EcoRI site is underlined.
azsF_F	TCGAAGGTAGGCATATGGAACCAGCCACCAGCTTC	NdeI site is underlined.
azsF_R	CGACAAGCTTGAATTCTCAGGCGGTGAGGTCCGC	EcoRI site is underlined.
azsO_F	TCGAAGGTAGGCATATGAGTCTCTTCGAGGTCCT	NdeI site is underlined.
azsO_R	CGACAAGCTTGAATTCTCATGAGGCACCGTCCTGGG	EcoRI site is underlined.
azsP_F	TCGAAGGTAGGCATATGACCGCCGCCCCCACCGG	NdeI site is underlined.
azsP_R	ATTCGGATCCCTCGAGTCAGGACGCATGGATGCCTT	XhoI site is underlined.
azsQ_F	TCGAAGGTAGGCATATGCGTCCTGACATCGAGCT	NdeI site is underlined.
azsQ_R	ATTCGGATCCCTCGAGTCAGACCGCCTTTCCGCTGT	XhoI site is underlined.
azsS_F	TCGAAGGTAGGCATATGAGCTGGAAGGACTTGGC	NdeI site is underlined.
azsS_R	ATTCGGATCCCTCGAGTCATCCGACGGTGTCCAGCC	XhoI site is underlined.
Sn_azsT_F	ATCGAAGGTAGGCATATGAGCGAGGGCGTGGGCAT	NdeI site is underlined.
Sn_azsT_R	CGACAAGCTTGAATTCCTACAAGGTGAAGACCTGGG	EcoRI site is underlined.
azsN_F	TCGAAGGTAGGCATATGATCATCCACAGGTACGAC	NdeI site is underlined.
azsN-CP_F	TCGAAGGTAGGCATATGGACGCCCGGGCCGAGGCCGA	NdeI site is underlined.
azsN-CP_R	CGACAAGCTTGAATTCTCATCGGTCCCCCTCGGCCG	EcoRI site is underlined.
azsO-CP_F	TCGAAGGTAGGCATATGACCCGCAACCGCAAGGTCGA	NdeI site is underlined.
AzsO-CP_R	CGACAAGCTTGAATTCTCATGAGGCACCGTCCTGGG	EcoRI site is underlined.

Table S3. The final concentration of IPTG for gene expression.

Protein	IPTG (mM)
AzsB	0.05
AzsD	0.05
AzsF	0.1
AzsN	0.1
AzsO	0.1
AzsP	0.05
AzsQ	0.1
AzsS	0.1
Sn_AzsT	0.05
AzsN-CP	0.1
AzsO-CP	0.1

Table S4. Primers used for site-directed mutagenesis of AzsO.

Name	Sequence (Changed nucleotides are indicated by underlining.)
azsOD193A_F	ACGTCAGCCACGCCGGCCTC
azsOD193A_R	CGGCGTGGCTGACGTGGAGC
azsOD198A_F	TCATGGCCGGCATCAGCATGT
azsOD198A_R	TGATGCCGGCCATGACGAGG
azsON414T_F	CACCTTCACCAGCGCCATCG
azsON414A_F	CACCTTCGCCAGCGCCATCG
azsON414A_R	AACGGCATCCTGGCGGCGCCCA
azsON447A_F	GCCGCCTTCGCGATGGAACAGCA
azsON447A_R	CATCGCGAAGGCGGCGAGCCACACTTGGGGGGTCT
azsON447D_F	GACGCCTTCGCGATGGAACAGCA
azsON447D_R	CATCGCGAAGGCGTCGAGCCACACTT

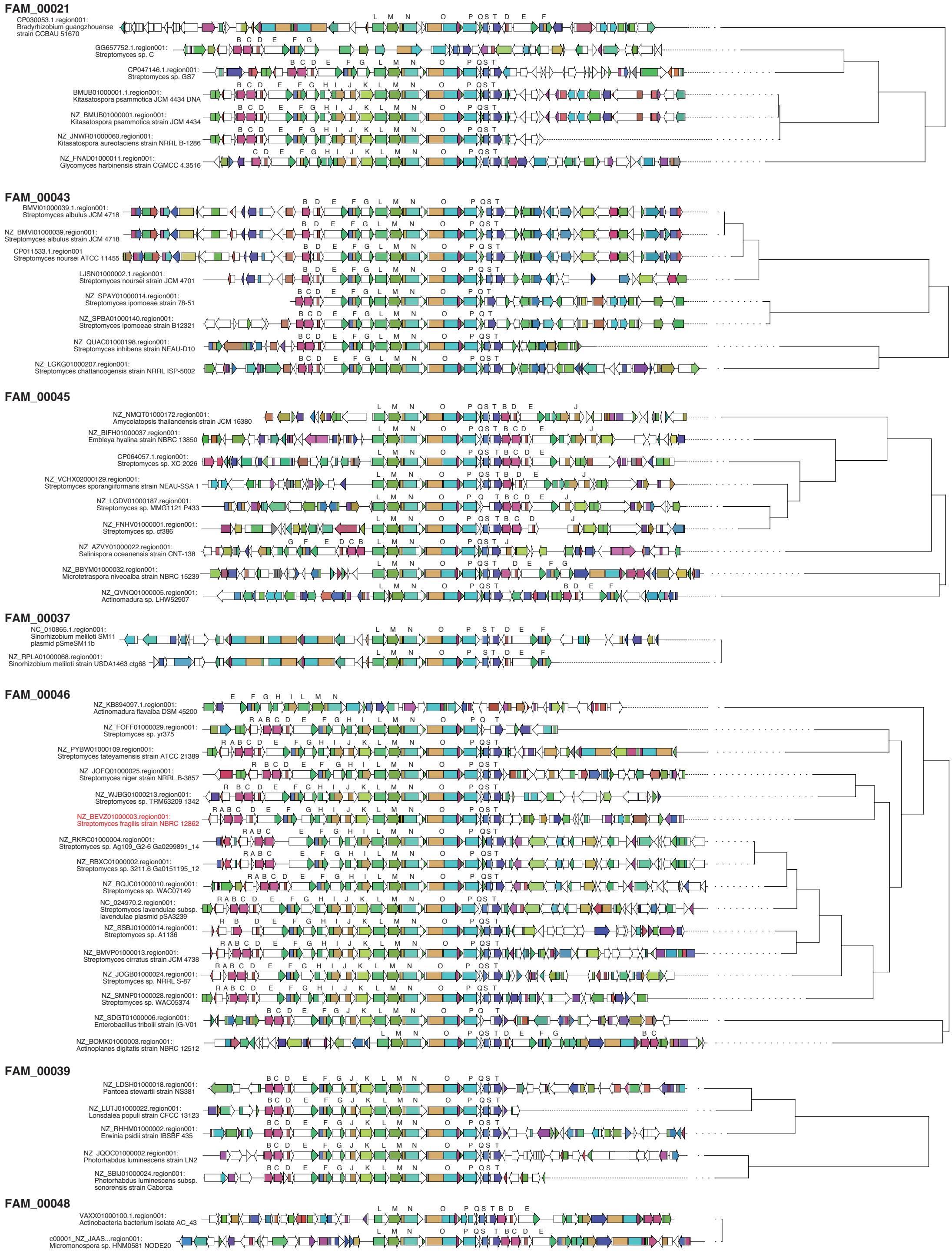

Figure S1. The *azs* cluster homologs discovered in the genome database. The clusters were analyzed by antiSMASH[4] and BiGSCAPE[5].

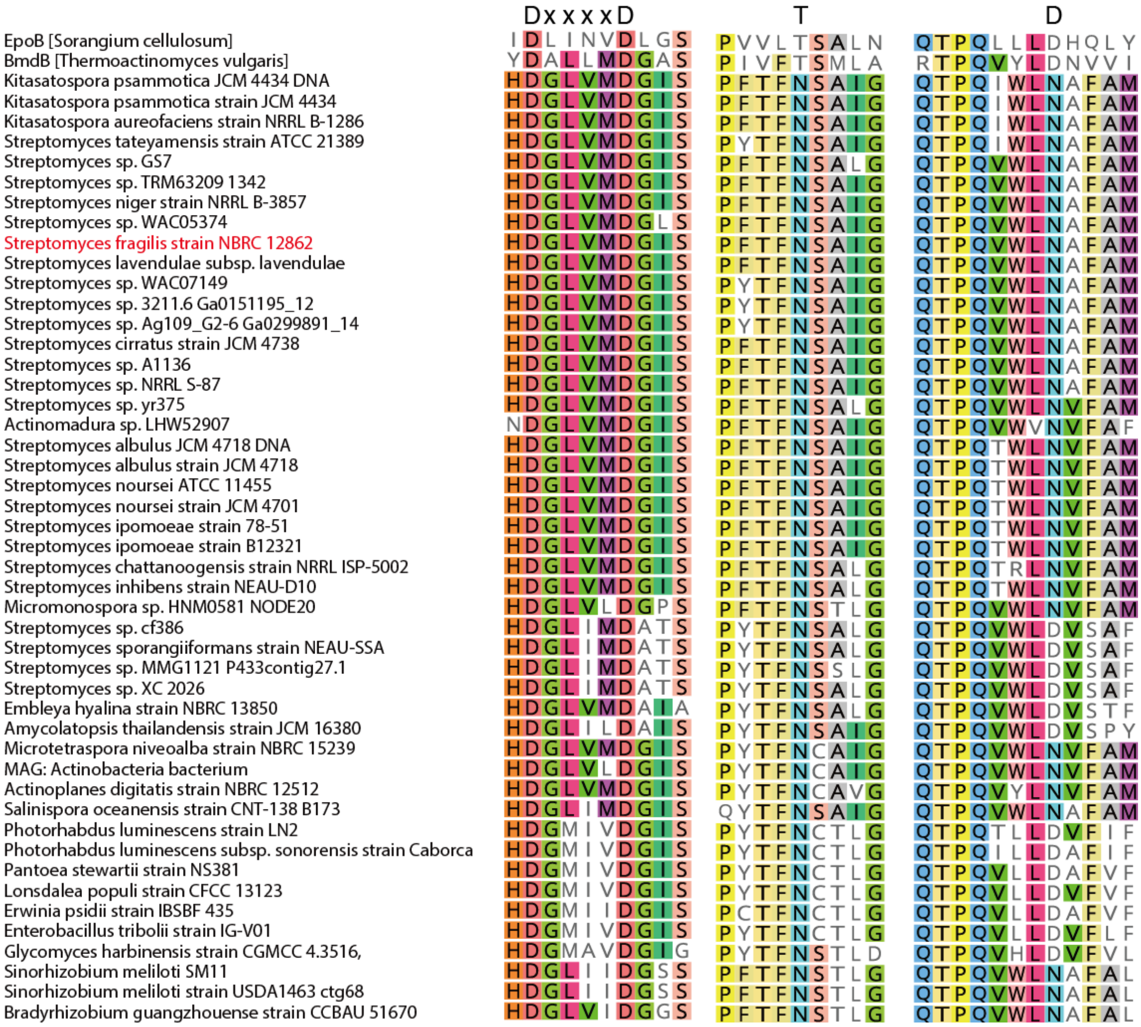

Figure S2. Amino acid sequence alignment of the Cy domains of AzsO homologs and representative Cy domains (EpoB and BmdB). The DxxxxD motif and two amino acid residues important for heterocyclization (Thr and Asp) are indicated on the top. The accession numbers of proteins used for alignment are as follows. PNE40067.1, QAU45219.1, TML31692.1, WP_009334107.1, WP_019872681.1, WP_030237850.1, WP_030596999.1, WP_033354816.1, WP_036806851.1, WP_051795528.1, WP_052868904.1, WP_053928179.1, WP_058702385.1, WP_067182460.1, WP_079432301.1, WP_091038154.1, WP_093779737.1, WP_093903507.1, WP_093938963.1, WP_094108101.1, WP_108953674.1, WP_053660137.1, GGU57162.1, GGX44379.1, ANZ21424.1, QHC22846.1, QQN76043.1, WP_012477297.1, WP_029727950.1, WP_110672440.1, WP_115457312.1, WP_117401004.1,WP_119101483.1, WP_121017858.1, WP_124231847.1, WP_125815249.1, WP_126641919.1, WP_126883298.1, WP_128509435.1, WP_136214109.1, WP_139656123.1, WP_141574023.1, WP_155059082.1, WP_169599406.1, WP_184995916.1, WP_189866485.1, and WP_190186751.1.

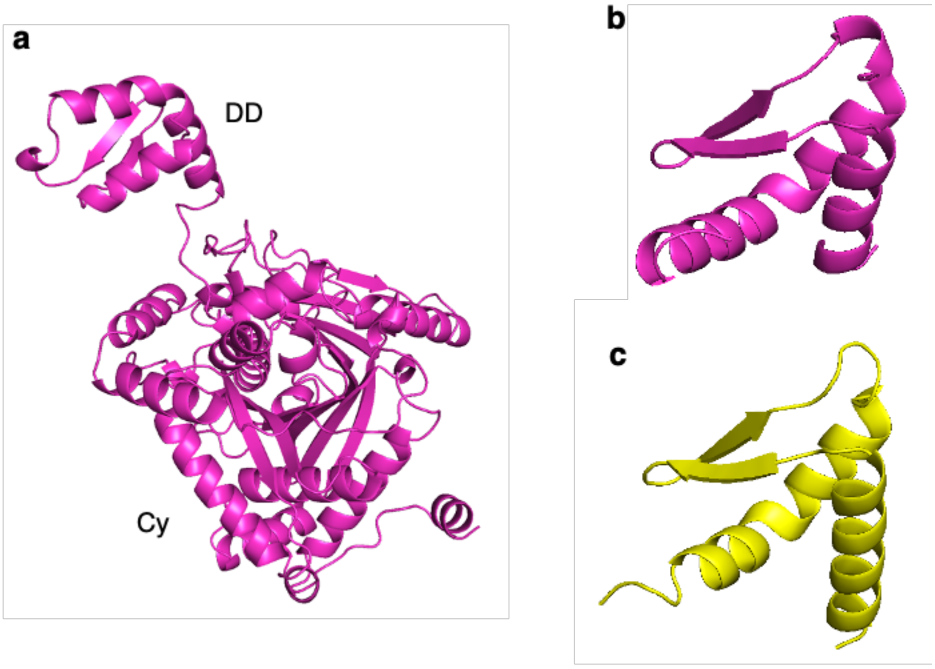

Figure S3. The structure models of AzsO from *Streptomyces fragilis* constructed by AlphaFold2. (**a**) The Cy domain of AzsO from *S. fragilis*. (**b**) The docking domain of AzsO from *S. fragilis*. (**c**) The docking domain of EpoB solved by X-ray crystallography. DD, docking domain; Cy, heterocyclization domain.

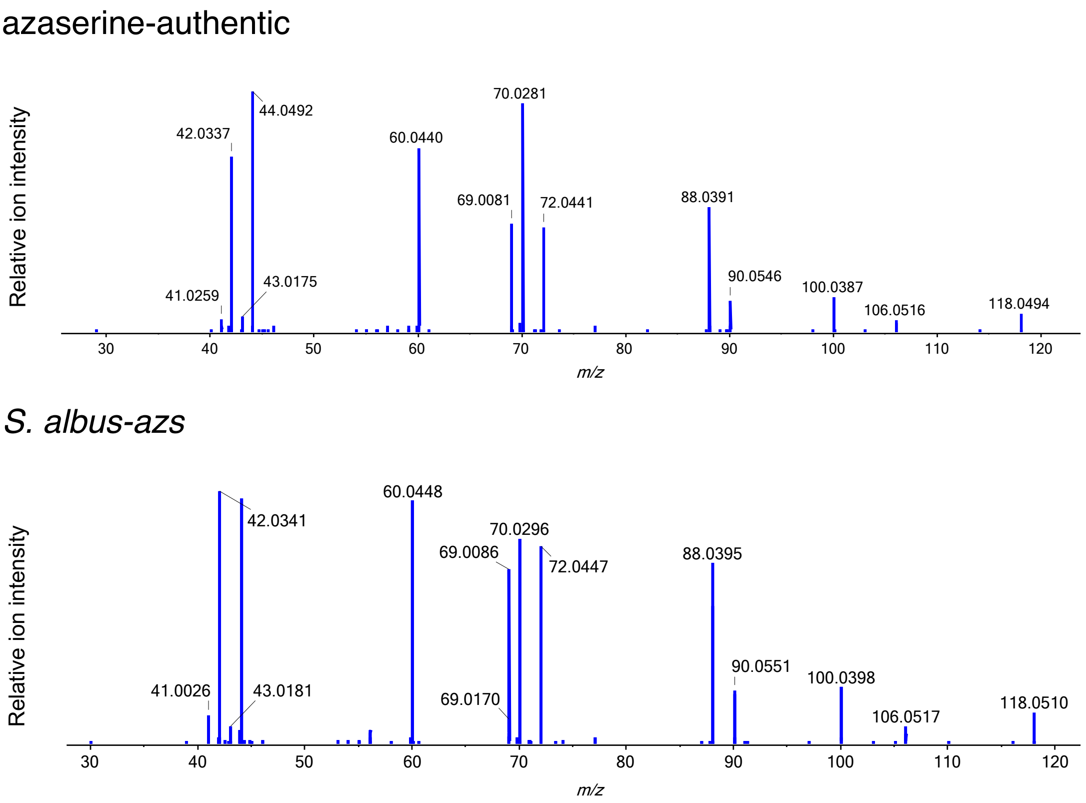

Figure S4. MS/MS spectra of authentic azaserine and the compound produced by *Streptomyces albus-azs*. MS/MS fragments are highly coincident between both compounds.

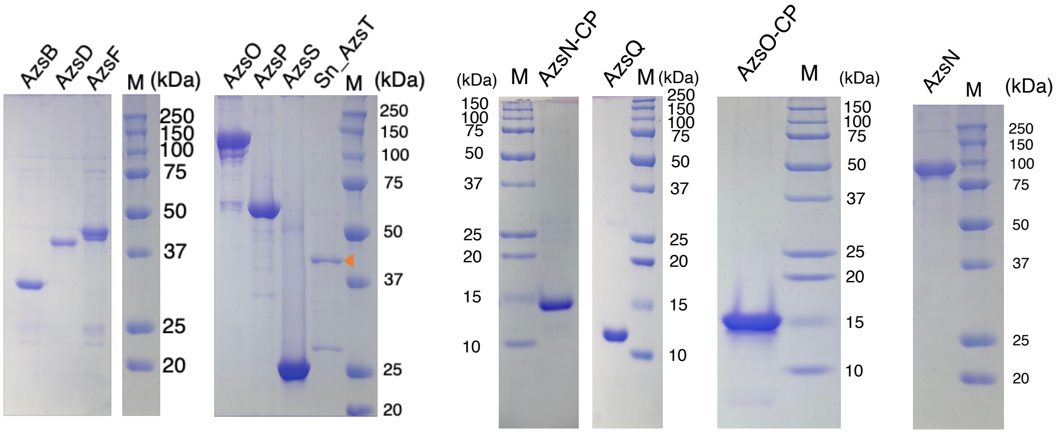

Figure S5. SDS-PAGE analysis of recombinant Azs proteins. The theoretical molecular mass of each recombinant protein is as follows. AzsB, 30.0 kDa; AzsD, 38.0 kDa; AzsF, 45.0 kDa; AzsO, 123.2 kDa; AzsP, 58.2 kDa; AzsS, 25.2 kDa; Sn_AzsT, 38.8 kDa; 45.0; AzsN-CP, 13.6 kDa; AzsO-CP, 13.8 kDa; AzsN, 85.5 kDa.

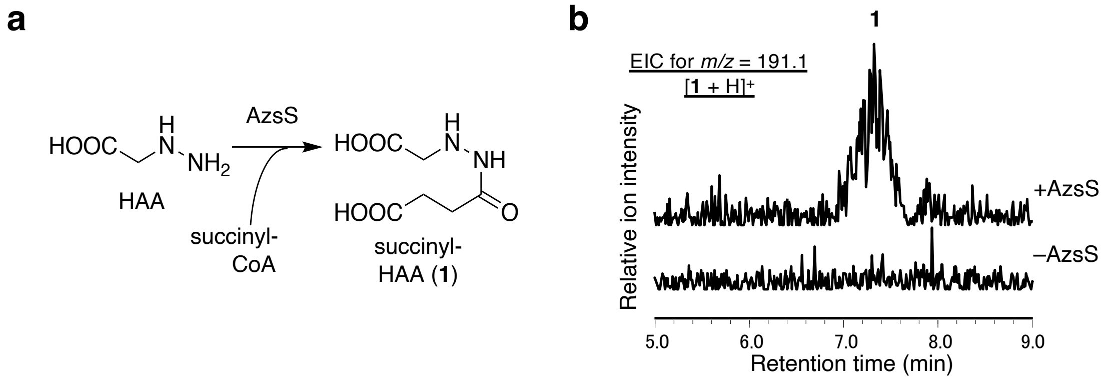

Figure S6. *In vitro* analysis of AzsS. (**a**) Overview of the pathway catalyzed by AzsS. (**b**) Extracted ion chromatograms of *m/z* 191.1, corresponding to $[M + H]^+$ ion of **1**.

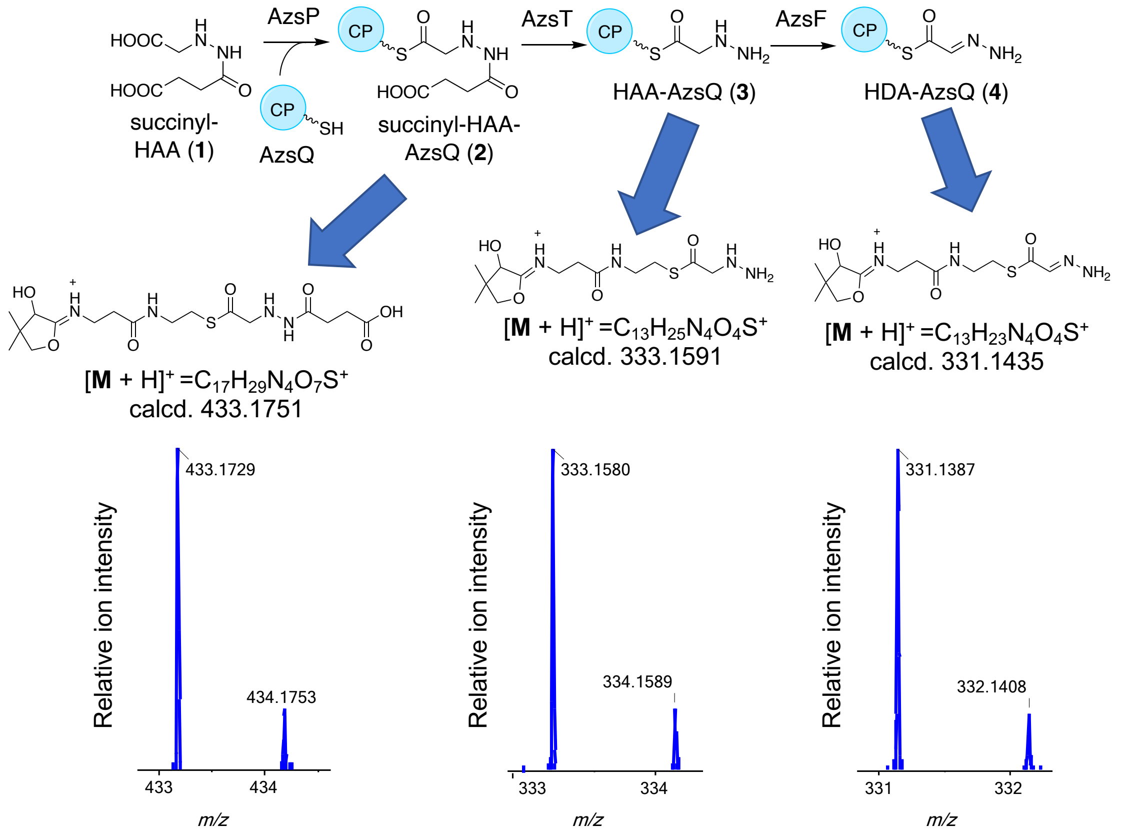

Figure S7. Phosphopantetheine ejection assay for the reaction products of AzsP, AzsT, and AzsF. The MS/MS fragment spectra obtained by the cleavage of phosphopantetheine moieties from **2**, **3**, and **4** are shown.

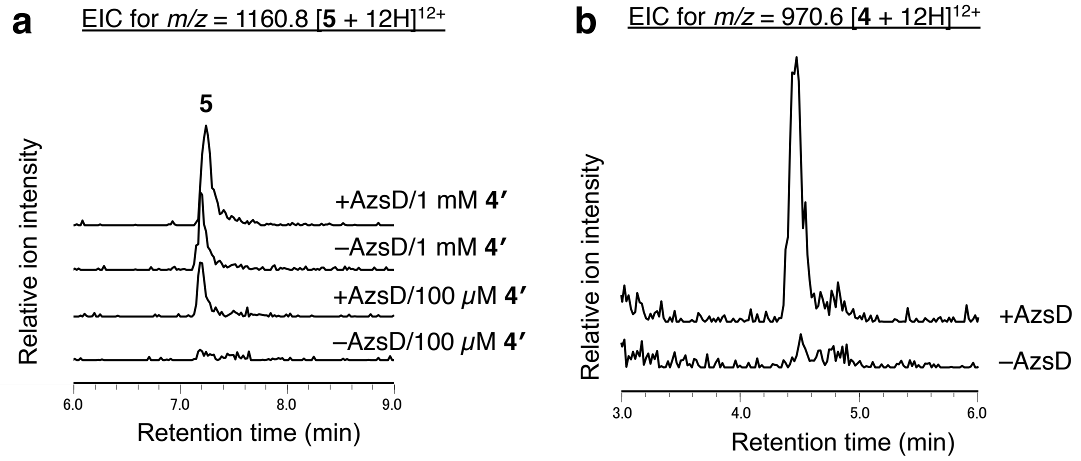

Figure S8. *In vitro* analysis of AzsD using HDA-NAC (**4′**) as a substrate. (**a**) LC-MS analysis of HDA-AzsN-CP (**5**) synthesized from HDA-NAC and AzsN-CP. The HDA moiety of HDA-NAC was spontaneously transferred to AzsN-CP when 1 mM of HDA-NAC was included. When the concentration of HDA-NAC was reduced to 100 μM, **5** was synthesized only in the presence of AzsD, indicating that AzsD can use HDA-NAC as a substrate. Extracted ion chromatograms of *m*/*z* 1160.8, corresponding to $[M + 12H]^{12+}$ ion of **5**, are shown. (**b**) LC-MS analysis of HDA-AzsQ (**4**) synthesized from HDA-NAC (100 μM) and *holo*-AzsQ. The HDA moiety of HDA-NAC was transferred to AzsQ only in the presence of AzsD. Extracted ion chromatograms of *m*/*z* 970.6, corresponding to $[M + 12H]^{12+}$ ion of **4**, are shown.

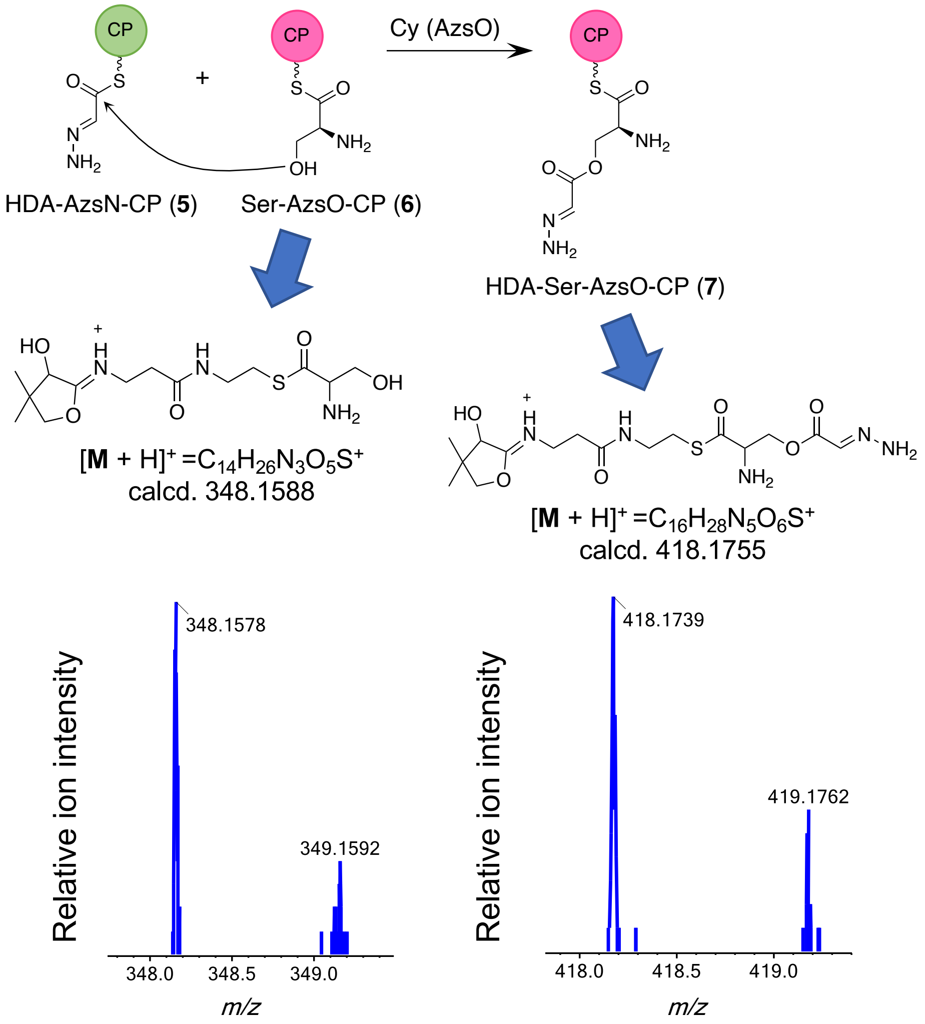

Figure S9. Phosphopantetheine ejection assay for the reaction product of AzsO. The MS/MS fragment spectra obtained by the cleavage of the phosphopantetheine moieties of Ser-AzsO-CP (**6**) and HDA-Ser-AzsO-CP (**7**).

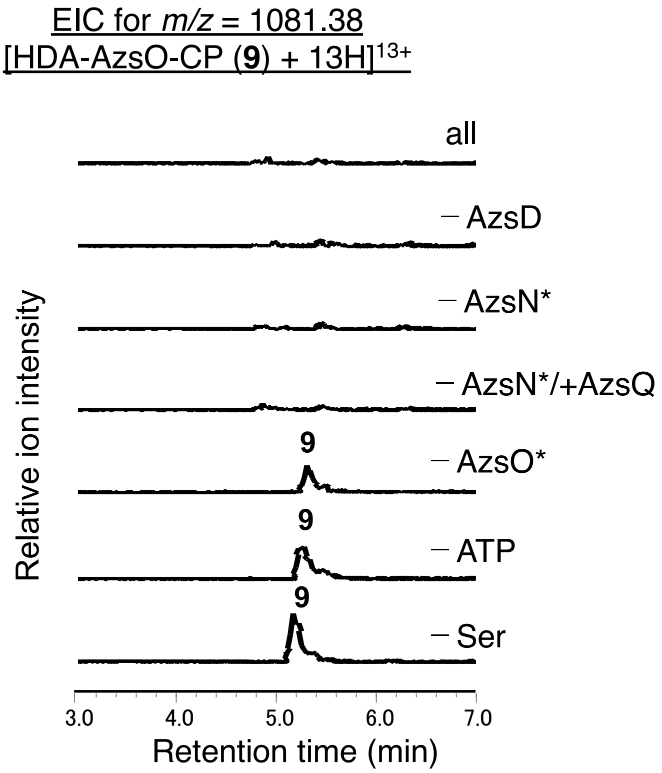

Figure S10. LC-HRMS analysis of HDA-AzsO-CP (**9**) in the reaction catalyzed by AzsD, AzsN, and AzsO. Extracted ion chromatograms of *m/z* 1081.38, which corresponds to $[M + 13H]^{13+}$ of **9**, are shown. AzsD can transfer the HDA moiety of HAD-NAC to the CP domain of AzsO only when L-serine is not attached to the CP domain of AzsO. The data indicate that AzsD can transfer HDA moiety nonselectively to various CPs. The proteins marked with * indicate the full-length proteins.

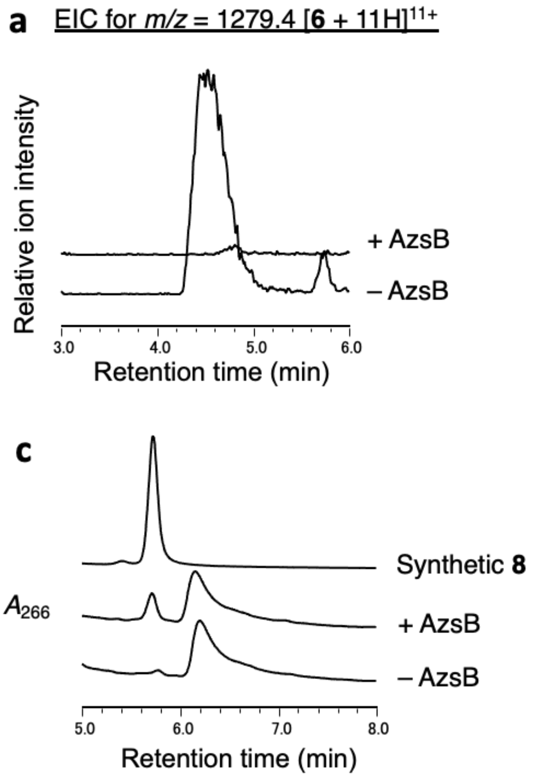

Figure S11. LC-MS analysis of HDA-Ser (**8**) release catalyzed by AzsB. (**a**) LC-MS analysis of Ser-AzsO-CP (**6**). Extracted ion chromatograms of *m/z* 1279.4, which corresponds to $[M + 11H]^{11+}$ of **6**, are shown. (**b**) LC-MS analysis of HDA-Ser-AzsO-CP (**7**). Extracted ion chromatograms of *m/z* 1285.7, corresponding to $[M + 11H]^{11+}$ ion of **7**, are shown. (**c**) Detection of HDA-Ser released from AzsO by AzsB. UV chromatograms at 266 nm are shown.

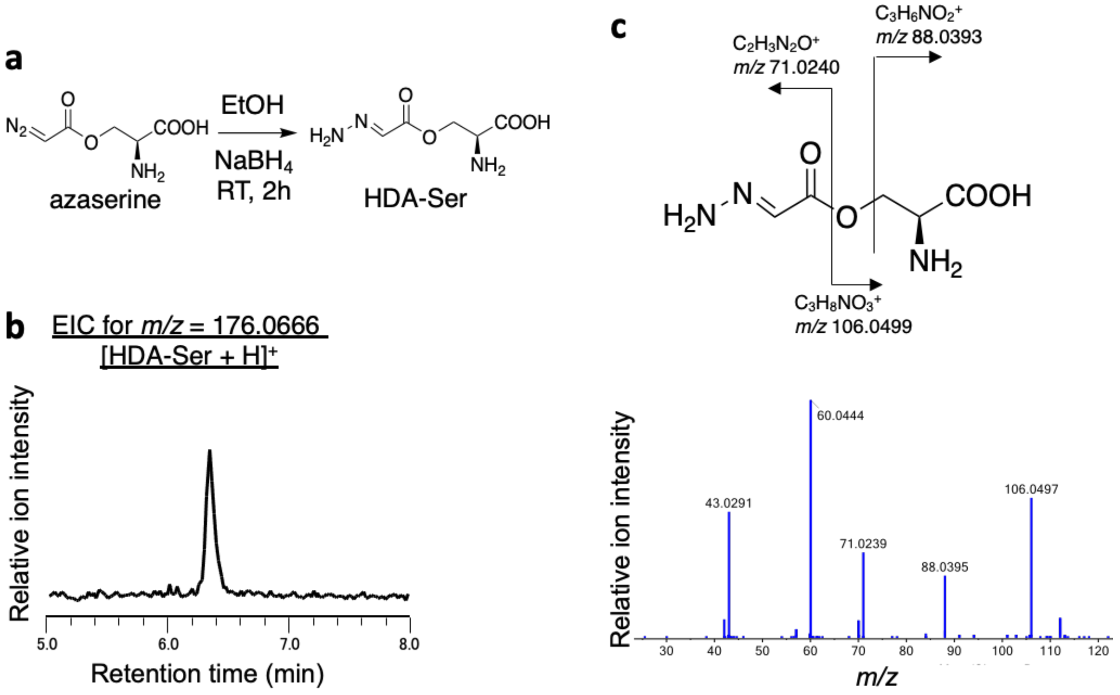

Figure S12. Synthetic standard of HDA-Ser. (**a**) Scheme of HDA-Ser synthesis. The diazo group of azaserine was reduced by sodium borohydride. (**b**) LC-HRMS analysis of HDA-Ser ($[M + H]^+ = C_5H_{10}N_3O_4^+$, *m/z* calcd. 176.0666, obs. 176.0665). (**c**) The MSMS spectrum of HDA-Ser. The important fragments are shown above the spectrum.

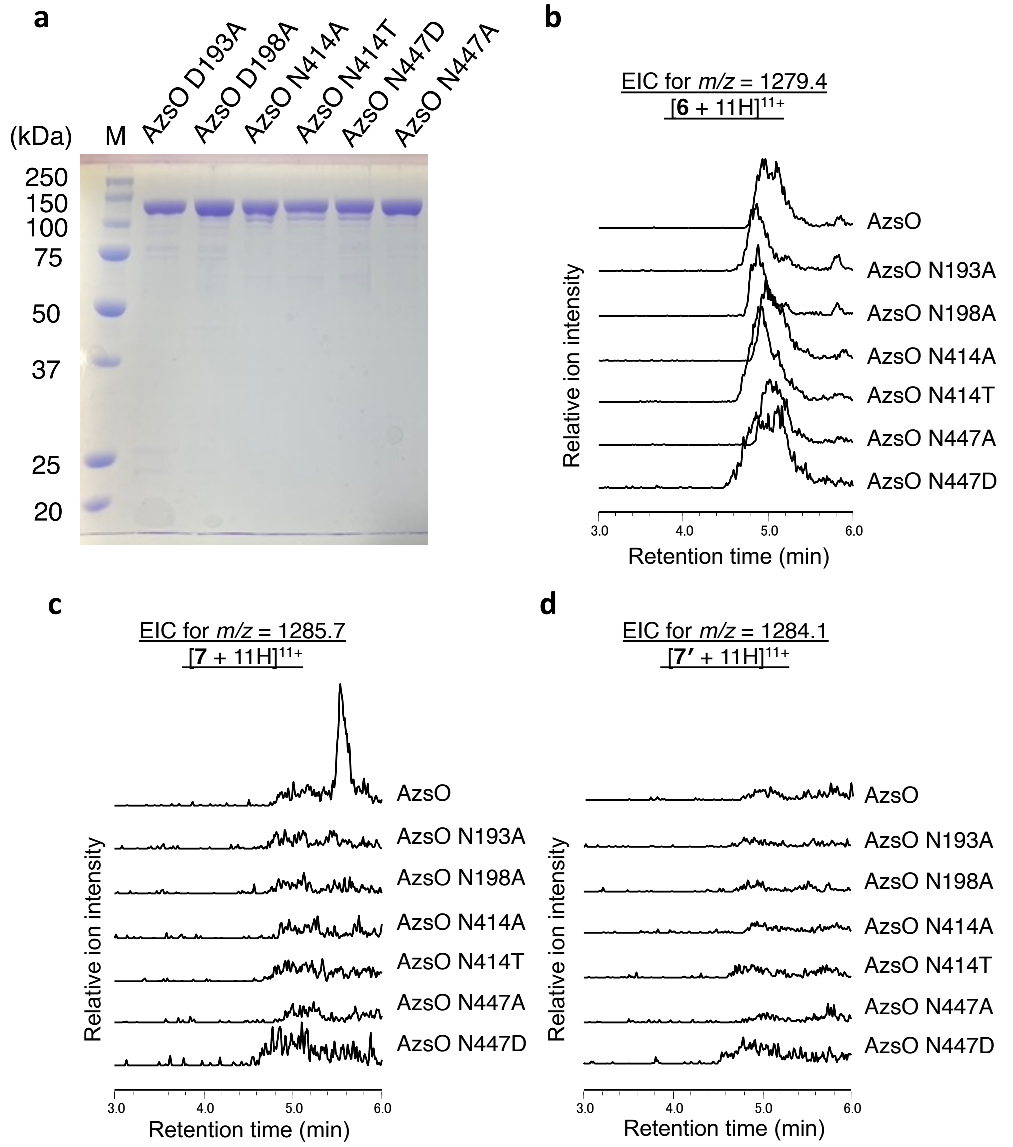

Figure S13. Site-directed mutagenesis of the Cy domain of AzsO. (**a**) SDS-PAGE of recombinant AzsO variants. (**b**) LC-MS analysis of Ser-AzsO-CP (**6**). Extracted ion chromatograms of *m/z* 1279.4, corresponding to $[M + 11H]^{11+}$ ion of **6**, are shown. (**c**) LC-MS analysis of HDA-Ser-AzsO-CP (**7**). Extracted ion chromatograms of *m/z* 1285.7, corresponding to $[M + 11H]^{11+}$ ion of **7**, are shown. Only wild-type AzsO catalyzed ester bond formation. (**d**) LC-MS analysis of cyclodehydrated HDA-Ser-AzsO-CP (**7′**). Extracted ion chromatograms of *m/z* 1284.1, corresponding to $[M + 11H]^{11+}$ ion of **7′**, are shown. The mutation did not result in cyclization ability. These results showed that these residues are important in catalysis. At least three independent replicates were performed for each assay. All the results showed same trends. Representative results are shown. WT, wild type.

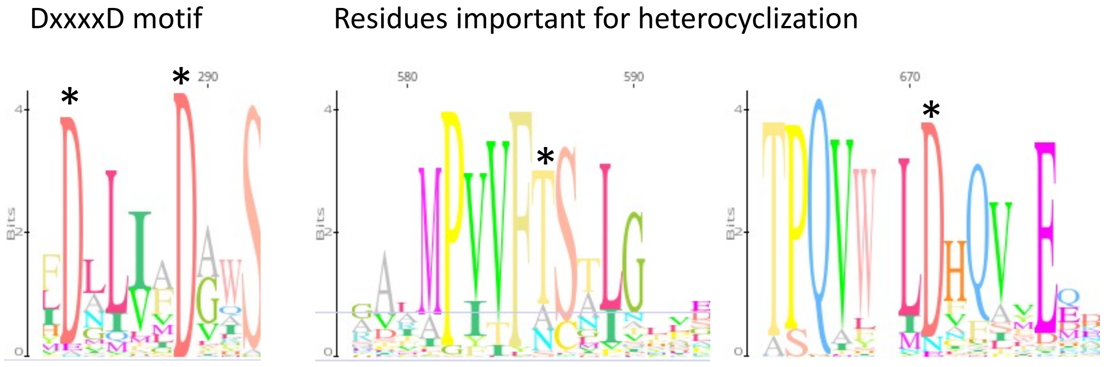

Figure S14. Sequence logo around conserved motifs generated from 1,165 Cy domains. The sequences of Cy domains were collected by the BLAST search using the Cy domain of AzsO as a query. The top 1,165 hits were used for generating the sequence logo. Two Asp residues of the DxxxxD motif, as well as the conserved Thr and Asp residues important for heterocycliztion, are marked with asterisks.

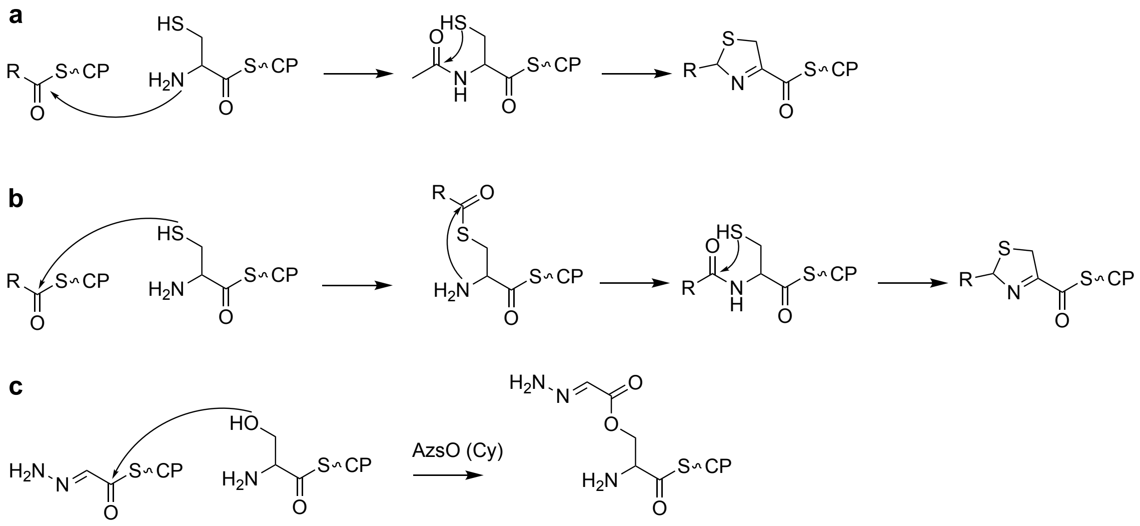

Figure S15. Mechanism of the reactions catalyzed by Cy domains. (a) The mechanism proposed generally. (b) The mechanism proposed by Shi *et al*. (c) The reaction catalyzed by the Cy domain of AzsO.

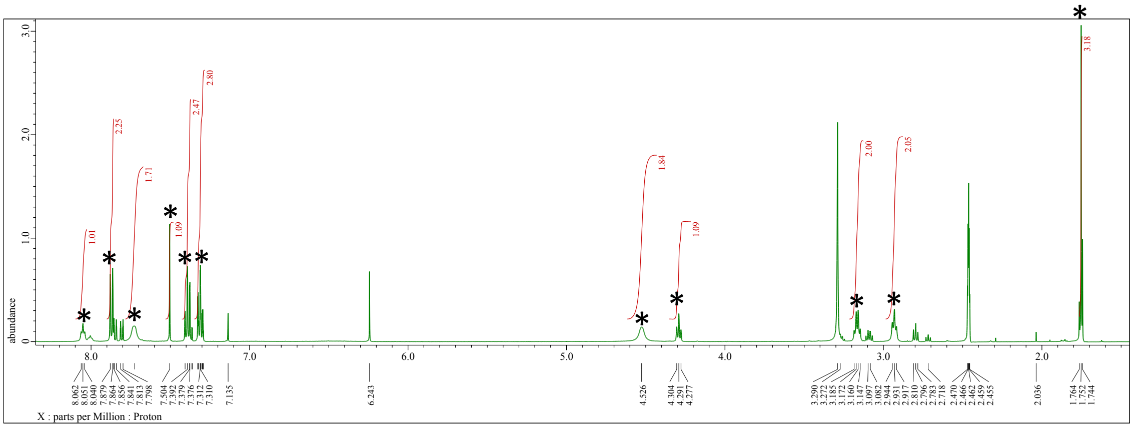

Figure S16. ^{1}H NMR data of Fmoc-HDA-NAC. Fmoc-HDA-NAC seems to be spontaneously hydrolyzed to Fmoc-HDA and *N*-acetylcysteamine. Therefore, degradation products are also detected in the chart. The signals corresponding to Fmoc-HDA-NAC are marked with asterisks.

References

[1] H. Onaka, S. Taniguchi, H. Ikeda, Y. Igarashi, T. Furumai, *J. Antibiot. (Tokyo)* **2003**, *56*, 950–956.

[2] T. Taguchi, T. Awakawa, Y. Nishihara, M. Kawamura, Y. Ohnishi, K. Ichinose, *Chembiochem Eur. J. Chem. Biol.* **2017**, *18*, 316–323.

[3] S. A. Malin, B. M. Laskin, A. S. Malin, *Russ. J. Appl. Chem.* **2007**, *80*, 2165–2168.

[4] K. Blin, S. Shaw, A. M. Kloosterman, Z. Charlop-Powers, G. P. van Wezel, M. H. Medema, T. Weber, *Nucleic Acids Res.* **2021**, *49*, W29–W35.

[5] J. C. Navarro-Muñoz, N. Selem-Mojica, M. W. Mullowney, S. A. Kautsar, J. H. Tryon, E. I. Parkinson, E. L. C. De Los Santos, M. Yeong, P. Cruz-Morales, S. Abubucker, A. Roeters, W. Lokhorst, A. Fernandez-Guerra, L. T. D. Cappelini, A. W. Goering, R. J. Thomson, W. W. Metcalf, N. L. Kelleher, F. Barona-Gomez, M. H. Medema, *Nat. Chem. Biol.* **2020**, *16*, 60–68.
